# A PPAR**γ**/long noncoding RNA axis regulates adipose thermoneutral remodeling in mice

**DOI:** 10.1172/JCI170072

**Published:** 2023-11-01

**Authors:** Zhengyi Zhang, Ya Cui, Vivien Su, Dan Wang, Marcus J. Tol, Lijing Cheng, Xiaohui Wu, Jason Kim, Prashant Rajbhandari, Sicheng Zhang, Wei Li, Peter Tontonoz, Claudio J. Villanueva, Tamer Sallam

**Affiliations:** 1 Division of Cardiology, Department of Medicine,; 2 Department of Physiology, and; 3 Molecular Biology Institute, UCLA, Los Angeles, California, USA.; 4 Division of Computational Biomedicine, Biological Chemistry, University of California, Irvine, Irvine, California, USA.; 5 Department of Pathology and Laboratory Medicine, UCLA, Los Angeles, California, USA.; 6 Diabetes, Obesity, and Metabolism Institute, Icahn School of Medicine at Mount Sinai, New York, New York, USA.; 7 Department of Biological Chemistry and; 8 Department of Integrative Biology and Physiology, College of Life Sciences, UCLA, Los Angeles, California, USA.

**Keywords:** Cardiology, Metabolism, Adipose tissue, Molecular genetics, Noncoding RNAs

## Abstract

Interplay between energy-storing white adipose cells and thermogenic beige adipocytes contributes to obesity and insulin resistance. Irrespective of specialized niche, adipocytes require the activity of the nuclear receptor PPARγ for proper function. Exposure to cold or adrenergic signaling enriches thermogenic cells though multiple pathways that act synergistically with PPARγ; however, the molecular mechanisms by which PPARγ licenses white adipose tissue to preferentially adopt a thermogenic or white adipose fate in response to dietary cues or thermoneutral conditions are not fully elucidated. Here, we show that a PPARγ/long noncoding RNA (lncRNA) axis integrates canonical and noncanonical thermogenesis to restrain white adipose tissue heat dissipation during thermoneutrality and diet-induced obesity. Pharmacologic inhibition or genetic deletion of the lncRNA *Lexis* enhances uncoupling protein 1–dependent (UCP1-dependent) and -independent thermogenesis. Adipose-specific deletion of *Lexis* counteracted diet-induced obesity, improved insulin sensitivity, and enhanced energy expenditure. Single-nuclei transcriptomics revealed that *Lexis* regulates a distinct population of thermogenic adipocytes. We systematically map *Lexis* motif preferences and show that it regulates the thermogenic program through the activity of the metabolic GWAS gene and WNT modulator TCF7L2. Collectively, our studies uncover a new mode of crosstalk between PPARγ and WNT that preserves white adipose tissue plasticity.

## Graphical abstract

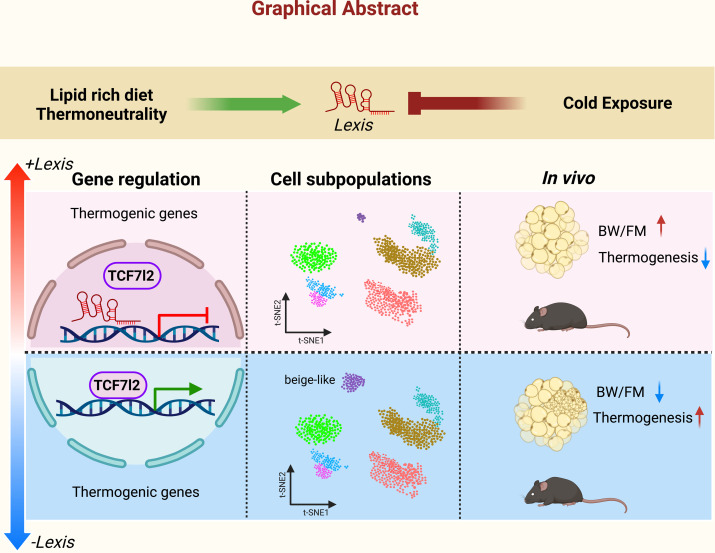

## Introduction

Adipose tissue plays a central role in maintaining metabolic homeostasis. White, beige, and brown adipocytes form the main parenchymal cells in adipose tissues (1–3). Brown adipocytes are multilocular cells found in brown adipose tissue (BAT) that dissipate heat in response to cold exposure, exercise, or β-adrenergic signaling (4). White adipose depots contain energy-storing white adipocytes as well as beige adipocytes, particularly enriched in subcutaneous or inguinal white adipose tissue (iWAT), which adopt a “brown”-like appearance and function (5). The mechanisms by which adipocytes adopt different fates have attracted substantial attention owing to their contributions to whole-body homeostasis and therapeutic implications for common metabolic diseases, such as obesity, metabolic syndrome, and diabetes (4).

Regardless of specialized function, all adipocytes require the activity of the master transcriptional regulator PPARγ for proper differentiation and function (6–8). Mice deficient in PPARγ have no adipose tissue, and PPARγ mutations in humans are associated with severe lipodystrophy (9–11). PPARγ binds PPAR response elements (PPREs), forming a permissive heterodimer with the retinoid X receptor (RXR), and upon ligand stimulation, its exchanges corepressors with coactivators to induce the expression of target genes essential for adipogenesis, maintenance of adipose tissue endocrine function, and thermogenic signaling (12–14). Given this established mode of transactivation, a mystery in the field is how PPARγ can preferentially activate a specific set of genes, but not others, depending on conditions. Thermogenic genes, for instance, are direct targets of PPARγ but not substantially enriched when PPARγ is potently activated by a lipid-rich diet (3, 15, 16). One level of tight regulation may be driven by cooperative transcriptional activity in response to environmental cues. For example, cold exposure activates PRDM16 and PGC1-a, which collaborate with PPARγ to specifically induce brown thermogenesis of a subset of cells in iWAT (17–19). However, it remains unclear how pathophysiologic PPARγ signaling during diet-induced obesity (DIO) or thermoneutral stimulation can specifically temper thermogenesis in white adipose depots. These observations hint that there may be milieu-specific regulatory circuits that fine-tune PPARγ responses. In addition, multiple lines of evidence suggest that classical activation of thermogenesis through uncoupling protein 1 (UCP1) upregulation may not be the sole mechanism responsible for heat dissipation (20, 21). For example, calcium signaling through ATP-dependent Ca^2+^ cycling by sarco/endoplasmic reticulum Ca^2+^-ATPase 2b (SERCA2b) as well as futile cycling of creatine metabolism contribute to UCP1-independent thermogenesis in beige fat (22, 23). Despite these exciting discoveries, the molecular pathways that integrate canonical and noncanonical thermogenesis during health and disease states remain poorly characterized.

Long noncoding RNAs (lnsRNAs) are defined as transcripts greater than 200 bp that do not make a protein product (24, 25). Rapidly accumulating evidence suggests that at least a subset of lncRNAs appears to be functional and to contribute to diverse biologic processes including metabolic control (26–28). Our previous work has shown that the lncRNA *Lexis* acts as a conduit between diet and cholesterol biosynthesis in liver (29). In response to lipid-rich feeding, *Lexis* is induced by liver X receptor (LXR), which in turn suppresses cholesterol biosynthesis by modulating the activity of the transcriptional coactivator RALY, but the majority of *Lexis* is expressed in extrahepatic tissues, where different isoforms with unknown function are enriched (29). A crucial advance that enhanced our understanding of lncRNA mechanisms and function has been the development of unbiased chromatin affinity assays that interrogate a lncRNA’s interactome (30–32). For example, identifying the binding patterns and interacting proteins of the lncRNA *Xist* illuminated key mechanisms involved in X inactivation and inspired approaches in investigating mechanisms of gene repression in physiology (33–35). Despite these advances, the systemic discovery of lncRNA targets has only been done for a handful of lncRNAs, and most reported lncRNA functional effects do not consolidate well with known physiologic adaptions. Here, we find that the lncRNA *Lexis* acts as a transcriptional brake for canonical and noncanonical thermogenic pathways in response to dietary and thermal cues. Identification of *Lexis*-associated genome-wide contact sites shows that this lncRNA targets thermogenic genes and SERCA2b through modulation of the transcriptional activator TCF7L2. Importantly, by performing extensive in vivo analysis in adipose-specific null mice, we demonstrate a causal link between *Lexis* and enrichment of specific thermogenic adipocytes. Collectively, these results provide evidence that beiging in WATs is “homeostatically” regulated via unique pathways that are not dependent on adaptive-thermogenic mechanisms.

## Results

### Adipose Lexis levels are dynamically regulated with diet and thermal stress.

**Figure 1:**
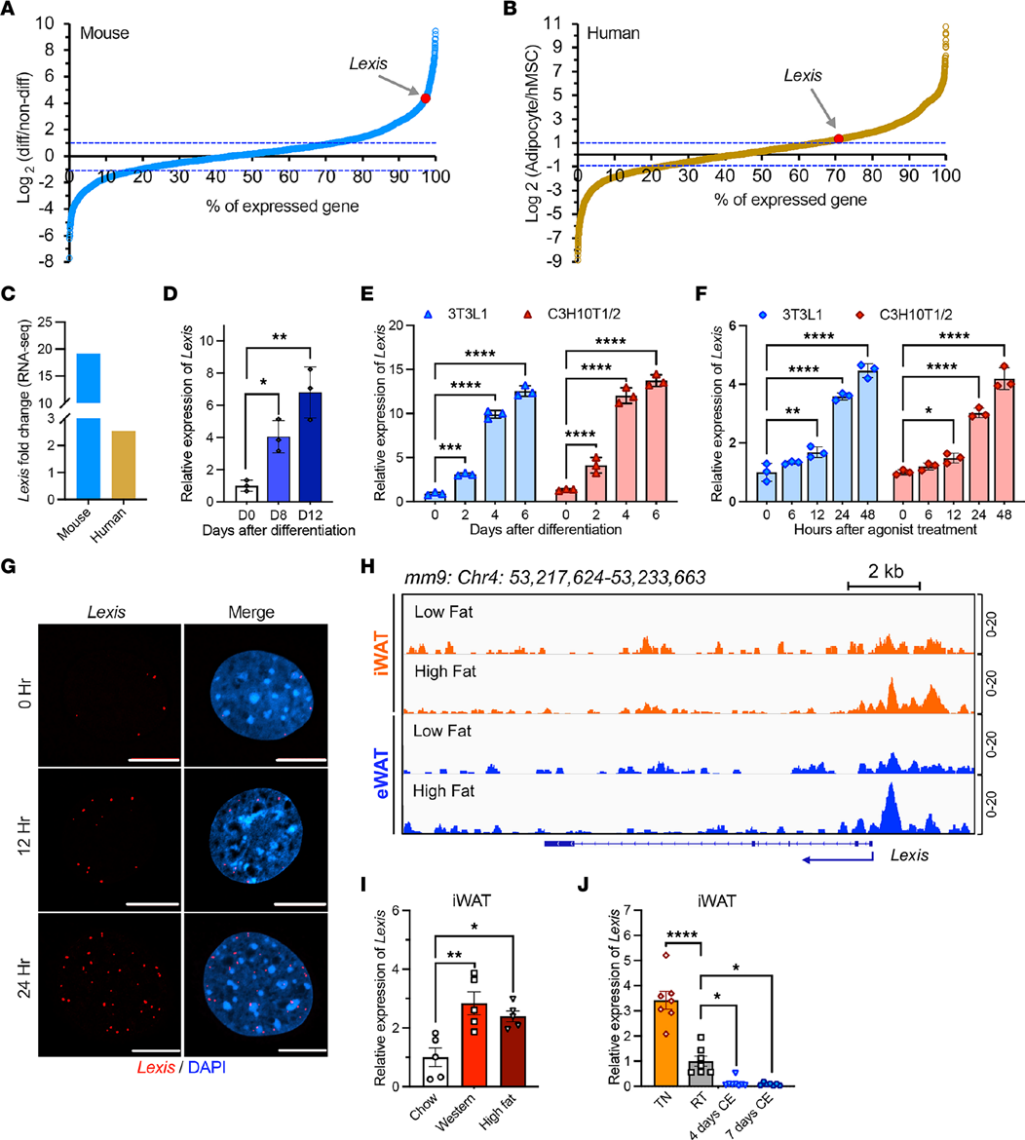
*Lexis* is regulated by PPARγ in adipose depots. (**A**) Fold change of lncRNAs in murine adipocytes after differentiation day 5 (Diff) compared with day 0 (non-diff) (from GEO GSE94654). (**B**) Fold change of lncRNAs in differentiated adipocytes (Diff) compared with nondifferentiated human mesenchymal stem cells (hMSCs) (from GEO GSE151324). (**C**) *Lexis* expression (human orthologue is putative) in differentiated versus nondifferentiated based on RNA-Seq data in **A** and **B**. (**D**) qPCR analysis in human adipose-derived mesenchymal stem cells (ADMSCs) (day 0) or differentiated adipocytes induced at day 8 or day 12 (*n* = 3 per group). (**E**) qPCR analysis of in C3H10T1/2 and 3T3L1 cells treated with differentiation cocktail (*n* = 3 per group). (**F**) qPCR analysis of C3H10T1/2 and 3T3L1 treated with PPARγ agonist GW1929 (20 nM) (*n* = 3 per group). (**G**) Single-molecule RNA-FISH targeting *Lexis* in C3H10T1/2 cells treated with vehicle or PPARγ agonist GW1929 (20 nM). Nuclear DNA was labeled with DAPI. Scale bars: 10 μm. (**H**) ChIP-Seq peaks of PPARγ iWAT and eWAT from GEO GSM2433426 (iWAT, low fat), GSM2433425 (iWAT, HFD), GSM2433449 (eWAT, low fat), and GSM2433453 (eWAT, HFD). (**I**) qPCR in iWAT from 10-week-old male mice placed on WD or HFD for 2 weeks (*n* = 5 per group). (**J**) qRT-PCR in iWAT from 8- to 10-week-old male mice under different thermal conditions, 7 days thermoneutrality (TN), 4 days cold exposure (CE), 7 days CE or room temperature (RT). *n* = 7 (TN, RT, and 7 days CE); *n* = 8 (4 days CE). Data are represented as mean ± SD (**D**, **E**, and **F**) and mean ± SEM (**I** and **J**). *P* values were calculated either by 1-way ANOVA (**D**, **I**, and **J**) or 2-way ANOVA (**E** and **F**). *P < 0.05; **P < 0.01; ***P < 0.001; ****P < 0.0001.

Tissue-wide gene expression analysis showed that the highest expression of endogenous *Lexis* is in adipose tissue (Supplemental Figure 1A; supplemental material available online with this article; https://doi.org/10.1172/JCI170072DS1). *Lexis* is more prominently expressed in white adipose depots, specifically in iWAT, compared with BAT (Supplemental Figure 1A). In addition, both our quantitative PCR and published data sets showed that the expression of *Lexis* is substantially higher in cells from WAT than BAT cellular origin, hinting that *Lexis* may play a regulatory role in white adipose depots (Supplemental Figure 1, B and C) (36). Analysis of published RNA-Seq data in mice and humans demonstrated that *Lexis* levels were elevated in differentiated adipocytes compared with nondifferentiated (Figure 1, A–C, and Supplemental Figure 1, D and E). Differentiation of human adipocytes showed enhanced *Lexis* expression along with adipose differentiation markers (Figure 1D and Supplemental Figure 1F). In addition, treatment of 2 different murine preadipocyte cell lines with differentiation cocktail or treatment with PPARγ agonist robustly induced *Lexis* expression along with established differentiation markers (Figure 1, E and F, and Supplemental Figure 1, G and H). Histone H3K27ac, a mark of active transcription, increased at the *Lexis* gene promoter region during adipocyte differentiation in both mice and humans, suggesting that the induction of *Lexis* during differentiation is primarily due to increased transcript biosynthesis (Supplemental Figure 1, I and J). Single-molecule FISH confirmed the induction of *Lexis* with adipocyte differentiation and showed that *Lexis* is primarily present in the nucleus (Figure 1G). Cellular fractionation analysis confirmed that the *Lexis* and Neat1 lncRNA are mostly present in the nucleus as opposed to the actin control transcript, which is predominantly found in the cytoplasm (Supplemental Figure 1K).

PPARγ is considered the master regulator of adipogenesis and WAT function (8, 13). Aligned with the high expression of *Lexis* in WAT and induction of *Lexis* during adipose differentiation, we identified PPARγ response elements within the *Lexis* promoter that were bound by PPARγ in ChIP-Seq studies from WAT (Figure 1H), suggesting that *Lexis* is a direct transcriptional target of PPARγ. To better explore the regulation of *Lexis* in vivo, we detected *Lexis* levels on WAT from mice fed chow, Western diet (WD), or high-fat diet (HFD) and under different temperatures. Quantitative PCR confirmed that *Lexis* is induced with lipid-rich diet feeding in iWAT (Figure 1I) as well as in mice exposed to thermoneutrality, but is reduced with cold exposure (Figure 1J). Collectively, these results suggest that adipose *Lexis* expression is tightly regulated in response to environmental cues and, at least in part, that this mode of regulation is driven by PPARγ.

### Genetic deletion or ASO inhibition of Lexis counteracts DIO.

**Figure 2:**
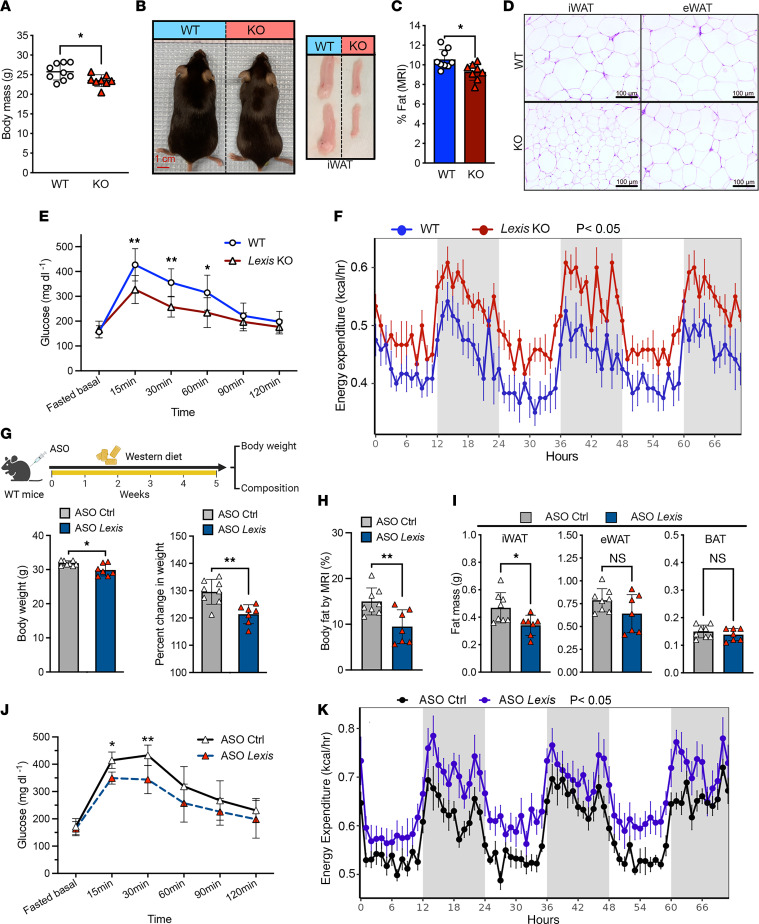
Loss of *Lexis* leads to lean phenotype. (**A**) Body mass after 3 weeks of WD feeding of *Lexis* WT or *Lexis-*KO mice (WT, *n* = 9; KO, *n* = 8). (**B**) Gross appearance and iWAT depot from WT or *Lexis-*KO mice. (**C**) Body fat composition determined by EchoMRI (WT, *n* = 9; KO, *n* = 8). (**D**) H&E staining of iWAT and eWAT from WT mice or *Lexis-*KO mice. Scale bars: 100 μm. (**E**) Glucose tolerance test (GTT) performed on male mice (*n* = 6 per group). (**F**) Energy expenditure from WT or *Lexis-*KO mice measured by indirect calorimetry (*P* = 0.0106, *n* = 6 per group). (**G**) Body weight and percentage changes of body mass from baseline of male mice treated with ASO control (Ctrl) or ASO *Lexis* placed on WD (Ctrl, *n* = 8; ASO *Lexis*, *n* = 7). (**H**) Body fat composition of mice in **G** determined by EchoMRI (Ctrl, *n* = 8; ASO *Lexis*, *n* = 7). (**I**) Fat depot mass after WD feeding (Ctrl, *n* = 8; ASO *Lexis*, *n* = 7). (**J**) GTT performed on mice after WD feeding (*n* = 9 per group). (**K**) Energy expenditure in ASO Ctrl or ASO *Lexis* mice using indirect calorimetry after WD feeding (*n* = 9 per group, *P* < 0.05 using either total body mass or lean body mass as covariates). Data in **A**, **C**, **E**, and **G**–**J** are represented as mean ± SD. Data in **F** and **K** are represented as mean ± SEM. *P* values were calculated by unpaired *t* test (**A**, **C**, **G**, **H**, and **I**) or by 2-way ANOVA (**E** and **J**). *P < 0.05; **P < 0.01; ***P < 0.001; ****P < 0.0001. Analysis of covariance (ANCOVA) was used for **F** and **K**.

To directly explore the contributions of endogenous *Lexis* in adipose tissue function in vivo, we fed *Lexis* global KO mice (*Lexis*-KO) or control mice (*Lexis* WT) WD. Compared with controls, *Lexis*-KO mice had lower body weight (Figure 2, A and B, and Supplemental Figure 2A) and percentage of fat (Figure 2C), but not lean weight, by MRI (Supplemental Figure 2B). Histologic appearance of iWAT showed smaller adipocytes in the *Lexis*-KO compared with *Lexis* WT, whereas epididymal WAT (eWAT) depots showed similar architecture between groups (Figure 2D). In addition, *Lexis*-KO mice showed improvement in glucose tolerance despite basal glucose levels similar to those of controls (Figure 2E). Loss of *Lexis* was not associated with changes in food intake or activity (Supplemental Figure 2, C and D), but resulted in an increase in energy expenditure (Figure 2F) and oxygen consumption (Supplemental Figure 2E).

Antisense oligonucleotides (ASOs) have been FDA approved or are in clinical trials for over 50 clinical diseases (37). We treated WT mice with *Lexis* ASO or control ASO (29) and fed them WD for 5 weeks (Figure 2H). *Lexis* ASO–treated mice showed lower expression of *Lexis* in iWAT (Supplemental Figure 2F) and a significant reduction in body weight from baseline compared with control ASO–treated mice (Figure 2G). Mice treated with *Lexis* ASO showed significantly lower percentage of fat by MRI without changing percentage of lean or body length (Figure 2H and Supplemental Figure 2, G and H). Fat-mass measurement indicated this difference was mainly from iWAT (Figure 2I). In addition, compared with control ASO, mice treated with *Lexis* ASO had improved glucose tolerance (Figure 2J) and enhanced energy expenditure (Figure 2K) without a change in food intake and activity (Supplemental Figure 2, I and J). Treatment of *Lexis*-KO mice with *Lexis* ASO or control ASO resulted in no differences in weight, confirming that the effects of ASO treatment on weight are specific to *Lexis* (Supplemental Figure 2K). Collectively, these results suggest that chronic or acute deletion of *Lexis* results in lower body weight, reduced fat mass, and improved glucose tolerance in response to a calorie-rich diet.

### Adipose-specific loss of Lexis alters global energy expenditure.

**Figure 3:**
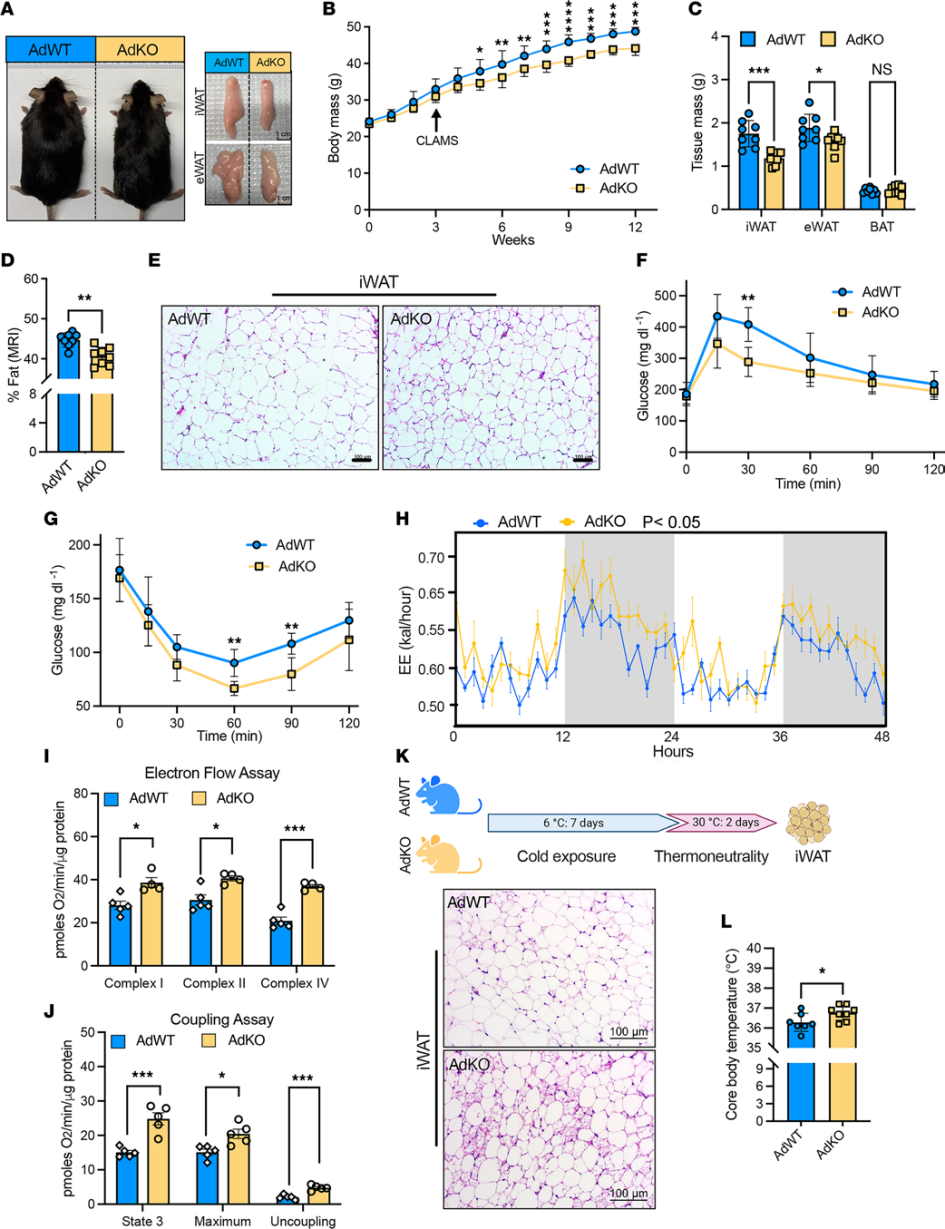
Adipose-specific deletion of *Lexis* counteracts DIO by increasing energy expenditure. (**A**) Gross appearance of *Lexis* adipose tissue–specific KO mice (AdKO) or littermate control mice (AdWT) and their adipose depots after 12 weeks of WD. (**B**) Body weight of AdKO or AdWT on WD (male, *n* = 8). (**C**) Fat pad mass after 12-week WD feeding (*n* = 8). (**D**) Body fat composition of 12-week WD-fed mice determined by EchoMRI (*n* = 8). (**E**) H&E staining of iWAT from mice in **B**. Scale bars: 100 μm. (**F**) Intraperitoneal glucose tolerance test administered on WD (male, *n* = 8). (**G**) Intraperitoneal insulin tolerance test on WD (Male, *n* = 8). (**H**) Energy expenditure (EE) using indirect calorimetry in male mice after 3 weeks on a WD, showing the mean value per hour ± SEM (*n* = 10, *P* < 0.05 by ANCOVA using either total body mass or lean body mass as covariates). (**I** and **J**) Average OCR in electron flow (**I**) and coupling Figure 3 (**J**) assays of mitochondria isolated from differentiated SVF from iWAT of AdKO or AdWT mice (**I**, *n* = 5 for AdWT, *n* = 4 for AdKO; **J**, *n* = 5 per group). (**K**) Schematic of experiment and H&E staining of iWAT. Scale bars: 100 μm. (**L**) Core body temperature measured from **K** (AdWT, *n* = 7, 5 male, 2 female; AdKO, *n* = 8, 5 male, 3 female). Data are represented as mean ± SD (**B**–**D**, **F**, **G**, and **L**) or mean ± SEM in Figure 2 (**H**, **I**, and **J**). *P* values were calculated by unpaired *t* test (**C**, **D**, **I**, **J**, and **L**) or 2-way ANOVA (**B**, **F**, and **G**). *P < 0.05; **P < 0.01; ***P < 0.001; ****P < 0.0001.

Although the above results hint that the observed body weight changes in *Lexis*-KO mice are likely due to altered systemic bioenergetics, they do not directly underpin adipose tissue as casually implicated. Notably, global loss of *Lexis* is associated with a mild fatty liver phenotype under a dietary challenge (29). To more directly explore the adipose-selective contribution of *Lexis*, we generated a *Lexis^fl/fl^* model and crossed it with adiponectin-cre to generate an adipose-specific loss of *Lexis* model (referred to as *Lexis*-AdKO or AdKO, and *Lexis^fl/fl^* Cre− referred to as *Lexis*-AdWT or AdWT) (Supplemental Figure 3A). Cre recombination ablated *Lexis* expression in adipose tissues, but not other metabolically active organs (Supplemental Figure 3B). Under chow conditions, *Lexis*-AdKO was not associated with a change in body weight, percentage of fat, or food consumption in male or female mice (Supplemental Figure 3, C–E). However, under a calorie-rich diet, adipose-specific loss of *Lexis* resulted in a lower body weight and reduced size and weight of white adipose depots, particularly iWAT (Figure 3, A–C). Consistent with these results, *Lexis*-AdKO mice had lower percentages of fat by MRI (Figure 3D), but no changes in body length, lean mass, or serum cholesterol level (Supplemental Figure 3, F–H). In addition, we did not observe differences in histologic appearance or gene expression in BAT (Supplemental Figure 3, I and J). Conversely, histology from iWAT from *Lexis*-AdKO showed reduced adipocyte hypertrophy compared with controls (Figure 3E). Consistent with our global loss of function and ASO treatment models, *Lexis*-AdKO had improved glucose tolerance (Figure 3F) and were more insulin sensitive compared with controls (Figure 3G). In addition, *Lexis*-AdKO showed enhanced energy expenditures without a change in activity when placed in metabolic chambers prior to weight separation (Figure 3H and Supplemental Figure 3, K and L). In line with the changes in organismal bioenergetics, differentiated stromal vascular fractions (SVFs) isolated from *Lexis*-AdKO or *Lexis*-AdWT mice showed increased oxygen consumption rates in electron flow and mitochondrial uncoupling assays (Figure 3, I and J). We did not observe sex differences as a consequence of adipose-specific loss of *Lexis*, since female mice also showed lower body weight, fat mass, and improved metabolic parameters in a DIO model (Supplemental Figure 3, M–P).

Our data indicate that adipose-selective *Lexis* expression regulates systemic energy expenditure and hint that the thermogenic program activation in iWAT may be responsible for the changes in *Lexis*-AdKO during DIO. Our data also strongly suggest that *Lexis* is tightly regulated in response to thermal conditions, with the highest levels of *Lexis* observed during thermoneutral exposure (Figure 1J). We, therefore, probed the influence of *Lexis* on adipose tissue function under thermoneutrality. We placed *Lexis*-AdKO or *Lexis*-AdWT mice under cold exposure for 1 week, a condition known to enhance WAT thermogenesis, followed by 2 days at thermoneutrality (Figure 3K). Histologic examination of iWAT from control mice, as expected, showed minimal beige adipocytes, whereas *Lexis*-AdKO iWAT showed a substantial increase in number of beige cells (Figure 3K). In addition, *Lexis*-AdKO mice had a higher core body temperature than *Lexis*-AdWT counterparts (Figure 3L). Congruent with these results, we treated WT mice with ASO control or ASO-*Lexis* prior to thermoneutral challenge. ASO-*Lexis*–treated mice showed a higher number of beige cells compared with ASO control mice (Supplemental Figure 3Q). It should be noted that loss of *Lexis* at 6 degrees had a minimal effect on thermogenic responses (not shown), perhaps consistent with the acute reduction in *Lexis* levels observed with cold exposure. Collectively, these results suggest that *Lexis* modulates whole-body energy expenditure through adipose-dependent mechanisms and provide evidence that dynamic changes in *Lexis* regulate adipose tissue beiging.

### Lexis is required for homeostatic thermogenic regulation in response to diet.

**Figure 4:**
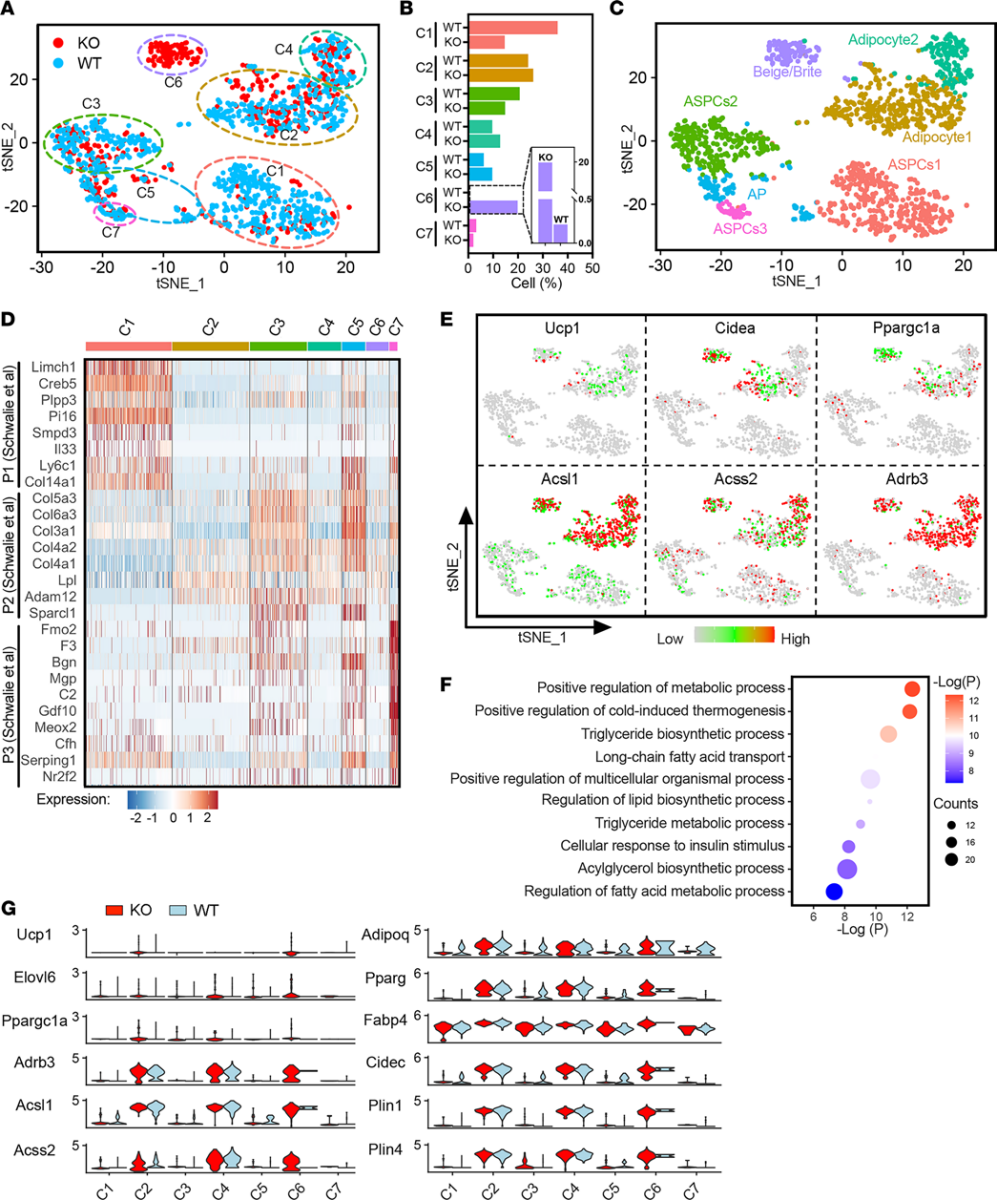
Loss of *Lexis* from adipose enriches for thermogenic populations. (**A**) t-Distributed stochastic neighbor embedding (tSNE) map of adipocyte-related subpopulation of adipose nuclei isolated from the iWAT of *Lexis*-AdWT mice (WT) and *Lexis*-AdKO mice (KO). (**B**) Fraction of WT and KO cells across each adipocyte-related subpopulation relative to the total number of adipocyte nuclei. (**C**) Annotation of adipocyte-related subpopulation derived from cluster-specific gene expression analysis (Supplemental Figure 3 G and Supplemental Table 1). (**D**) Heatmap showing average expression of genes as population markers identified by Schwalie et al., (43) in our identified adipocyte subpopulations. (**E**) tSNE plots highlighting the expression of representative thermogenic genes in adipocyte subclusters. (**F**) Function annotation of C6 subcluster in **B**. Top 10 functional terms (GO Biological Process) shown. (**G**) Normalized expression in different adipocyte subclusters under WT or KO group.

To further explore how loss of *Lexis* enhanced energy expenditure and can influence thermogenic capacity of WAT, we performed single-nucleus RNA-Seq (snRNA-Seq) on iWAT from *Lexis*-AdKO mice and *Lexis*-AdWT (WT) using recently established isolation strategies (Supplemental Figure 4A) (38, 39). Integrated transcriptomic analysis revealed 9 distinct population clusters (Supplemental Figure 4, B and C, and Supplemental Table 1). We annotated these populations using the marker genes according to previous publications and established data sets such as “PanglaoDB” and “CellMarker” (40, 41). Identified clusters included most of the major cell types known to be present in WAT by snRNA-Seq strategies including immune cells, adipocytes, adipocyte precursors, and endothelial cells (38). Frequency analysis in each cluster revealed that the major changes between WT and KO were driven by immune- and adipocyte-related populations (Supplemental Figure 4E). Considering that the dominant expression of *Lexis* is in adipocytes and that our previous studies showed that *Lexis* is not expressed in immune cells (42), we reasoned that the immune cell changes are likely a secondary effect of loss of *Lexis*. We therefore merged the adipocyte-related populations for further downstream analysis. Reclustering of these nuclei resulted in 7 distinct adipocyte-derived subpopulations (clusters C1 to C7) (Figure 4, A and B). Classical adipocyte marker genes *Fabp4* and *Adipoq*, but not the immune cell markers (*Mrc1* and *Ikzf1*), EC markers (*Cyyr1*), or myocyte markers (*Cacng1*), were highly expressed in all of these 7 subpopulations, indicating the specificity of adipocyte population selection (Supplemental Figure 4F). We annotated clusters using gene expression markers to segregate mature adipocytes, progenitor cells, and thermogenic/beige populations (Supplemental Figure 4G and Figure 4, B–D) to better understand what underlies population differences due to loss of *Lexis*. Interestingly, 3 of our identified cell clusters matched previously described adipogenic stem and precursor cells (ASPCs) isolated from mouse subcutaneous tissue (Figure 4, C and D). (43). The most dominant differences between WT and KO were noted in population C6 (Figure 4B), which was highly enriched in the KO group and expressed relatively high levels of thermogenesis-related genes including Ucp1, Cidea, Pgargc1a, Acsl1, Acss2, and Adrb3 without marked changes in Adipoq, Pparg, Fabp4, Cidec, Plin1, and Plin4 (Figure 4E). Notably, *Acsl1* and *Adrb3* were key markers of thermogenic “beige” adipocyte populations previously identified by snRNA-Seq in iWAT (44). Furthermore, pathway analysis of C6-positive genes revealed enrichment of thermogenesis-related pathways (Figure 4F) as well as higher expression of *Ucp1*, *Elovl6*, *Ppargc1a*, *Adrb3*, *Acsl1*, and *Acsl2* in the KO compared with WT (Figure 4G). Intriguingly, other populations expressing thermogenic genes (C2 and C4) were not altered between WT and KO, suggesting that *Lexis* does not “globally” influence all cells with thermogenic propensity in iWAT. In agreement with the physiologic studies, these results suggest loss of *Lexis* enriches specific thermogenic cells to enhance energy expenditure and strongly imply that basal thermogenesis in iWAT is tightly regulated, even in the absence of sustained β-adrenergic stimulation. 

### Systemic mapping of Lexis-binding sites reveals a direct role in canonical and noncanonical thermogenesis.

**Figure 5:**
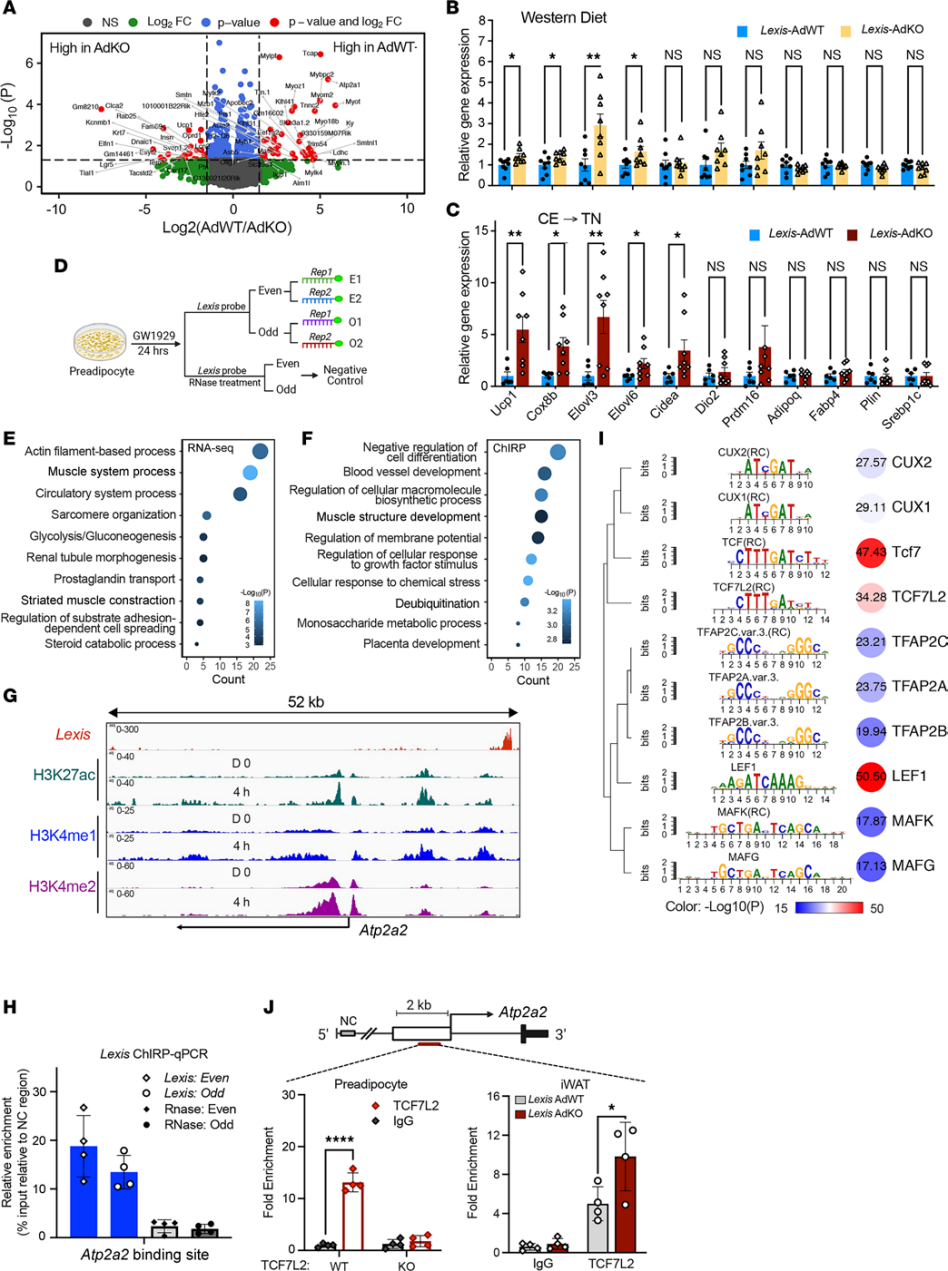
Systemic identification of *Lexis “*interactome” shows direct contact with Atp2a2 promoter region. (**A**) Volcano plot of RNA-Seq data in the iWAT of mice fed 12-week WD (*n* = 3 per group). Red dots indicate the gene highly expressed in AdWT or AdKO group (Cutoff: fold change [FC] > 1.5 and *P* < 0.05). (**B**) Gene expression by qRT-PCR in the iWAT of 12-week WD-fed mice in Figure 3 (**B**) (*n* = 8 per group). (**C**) Gene expression by qRT-PCR in the iWAT of mice given temperature stress in Figure 3 (**I**) (*n* = 6 for *Lexis*-AdWT; *n* = 8 for *Lexis*-AdWT). (**D**) Experimental schematic *Lexis* chromatin-affinity assay. (**E**) Top enriched pathways analyzed by Metascape using the data from *Lexis* RNA-Seq in **A**. (**F**) Top enriched pathways analyzed by Metascape using the data from **D**. (**G**) Representative peaks of *Lexis*, H3K27ac ChIP-Seq, H3K4me1 ChIP-Seq, and H3K4me2 ChIP-Seq on Atp2a2 promoter region. *Lexis* peaks are from this experiment and other ChIP-Seq from data sets under GEO GSE56872 and GSE95533. (**H**) *Lexis* ChIRP-qPCR using primers targeting binding sites of *Lexis* at Atp2a2 in 10T1/2 cells treated with 24 hours of GW1929 (*n* = 4 per group). (**I**) Motif analysis based on *Lexis*-enriched contact sites. The values in the circles revealed the −log_10_ (*P* value). (**J**) TCF7L2 ChIP-qPCR performed in TCF7L2 WT or TCF7L2 KO preadipocytes (left) or in iWAT of *Lexis* WT (*Lexis* AdWT) or *Lexis*-KO (*Lexis*-AdKO) mice with 1 week of thermoneutrality (*n* = 4 per group). Data are represented as mean ± SD (**H** and **J**) or mean ± SEM (**B** and **C**). *P* values were calculated by unpaired *t* test (**B**, **C**, and **J**). *P < 0.05; **P < 0.01; ****P < 0.0001.

To further explore the mechanism by which *Lexis* affects adipose tissue function, we performed bulk RNA-Seq of iWAT from *Lexis*-AdKO mice and *Lexis*-AdWT mice. Analysis of 3 biological replicates from each group identified 200 significantly differential expressed genes (*P* < 0.05), with 99 genes upregulated and 101 genes downregulated in response to adipose tissue–specific deletion of *Lexis* (Figure 5A). While the vast majority of genes were similarly expressed, genes linked to adipocyte thermogenesis, such as *Ucp1* and *Cox8b*, were elevated in iWAT from *Lexis*-AdKO mice compared with controls (Figure 5A). We further confirmed this result by quantitative reverse transcriptase PCR (qRT-PCR) where loss of *Lexis* enhanced *Ucp1* and thermogenic gene expression in a DIO model (Figure 5B). Furthermore, the gene expression changes were significantly more pronounced during the transition from cold exposure to thermoneutrality (Figure 5C). Loss of *Lexis* did not have a significant effect on the beiging regulator *Prdm16* or canonical whitening genes (Figure 5C). Finally, comparative analysis of global gene expression with published data sets (NCBI’s Gene Expression Omnibus databse; GEO GSE8044) (45) indicated that the *Lexis*-AdKO signature more strongly overlapped with BAT rather than WAT (Supplemental Figure 5A).

*Lexis* is predominantly found in the nucleus and specifically associates with chromatin, which led us to hypothesize that *Lexis* may affect thermogenic gene expression through altering epigenetic landscape or transcriptional dynamics. To investigate this further, we used an unbiased chromatin affinity assay that allowed us to map *Lexis*-dependent genome contact sites (30, 46). This approach is analogous to a ChIP for a transcription factor and has been used to decipher key molecular activities of other lncRNAs (30, 33, 47). We generated 2 sets of *Lexis* pulldown library probes, “even” and “odd” probes, and RNAase control groups to ensure that the RNA-pulldown signal was *Lexis* specific (Figure 5D). The qRT-PCR analysis of the retrieved RNA showed that 63.2% of the endogenous *Lexis* was specifically pulled down by 2 different *Lexis* probe sets (Supplemental Figure 5B). As negative control, the highly abundant RNA 18S rRNA and another confirmed nuclear-enriched lncRNAs *Mexis* were undetectable in *Lexis* chromatin isolation by RNA purification (ChIRP) samples (Supplemental Figure 5B), confirming the specificity of the probes to *Lexis* (42, 48). In addition, RNase treatment abolished the retrieval of *Lexis* or other lncRNAs, indicating that *Lexis* detection in our samples was RNA dependent (Supplemental Figure 5B). Leveraging this approach, we mapped genome-wide *Lexis*-binding sites (Supplemental Figure 5, C and D). Notably, *Lexis* bound genes involved in mitochondrial metabolism/thermogenesis, fatty acid regulation, and glucose metabolism (Supplemental Figure 5E). We performed Metascape pathway analysis using differential expressed genes from RNA-Seq and top enriched genes from *Lexis* probe retrieval (49). In line with the gene expression data and in vivo phenotyping, metabolic terms such as “Glycolysis/Gluconeogenesis” and “Steroid catabolic process” were enriched, but we also noted strong enrichment of pathways related to muscle development and function in both the RNA-Seq and ChIRP (Figure 5, E and F). Although beige adipocytes are not uniform in origin and function, some are thought to arise from myogenic-like cells (50). More recently, it has been shown that canonical genes involved in muscle function, including those involved in Ca^2+^ cycling, can drive UCP1-independent beige fat thermogenesis (23, 51). In addition, optogenetic stimulation of Ca^2+^ cycling fat thermogenesis in iWAT increases whole-body energy expenditure (52). Unexpectedly, we identified *Lexis* binding peaks in the genomic region of several general Ca^2+^ cycling target genes including *Atp2a2* and *PrKaa1*, suggestive of direct interactions between *Lexis* and the Ca^2+^ cycling signaling pathway (Figure 5G and Supplemental Figure 5F). Thus, we reasoned that, in addition to affecting canonical thermogenesis, *Lexis* could directly target the Ca^2+^ cycling pathway to affect heat dissipation. Among the genes involved, *Atp2a2* specifically caught our attention because its encoding protein SERCA2b is the key regulator of UCP1-independent beige fat thermogenesis signaling (23, 51) and *Atp2a2* had a one of the top-enriched *Lexis* binding peaks (Figure 5G and Supplemental Figure 5D). Cardiac muscle function or development was also enriched in analysis of deposited gene expression data sets of UCP1-independent thermogenic activation (Supplemental Figure 5G). *Lexis* binds upstream of the *Atp2a2* transcription start site (TSS) (Figure 5G). Interestingly, computational analysis using “LongTarget” predicted DNA binding for *Lexis* at *Atp2a2* (53, 54) near the ChIRP site (Supplemental Figure 5H). In addition, we performed ChIRP-qPCR to verify RNA-chromatin interactions between the *Lexis* and *Atp2a2* genomic regions (Figure 5H) (31). Intriguingly the *Lexis* binding site is near H3K27ac, H3K4me1, and H3K4me2 histone marks, which are associated with the higher activation of transcription of nearby genes (Supplemental Figure 5G) (55, 56).

To identify the key transcriptional regulators affected by *Lexis*, we performed genome-wide motif analysis of *Lexis*-enriched binding sites from the chromatin pulldown assays. Unbiased interrogation revealed high enrichment of the closely related T cell factor/lymphoid enhancer binding factor family (TCF/LEF) members, including TCF7, TCF7L2, and LEF (Figure 5I). TCF7L2, a downstream transcriptional effector of the Wnt/β-catenin signaling pathway, has been extensively associated with metabolic disease in human genetic studies (57–60). Specifically, variants at TCF7L2 were found to have the strongest association with type II diabetes in human GWAS (61). Adipose tissue–specific deletion of TCF7L2 led to increased adipose mass and body mass in the response to HFD, the opposite phenotype of *Lexis* knockout mice (62–65). Binding patterns of TCF7L2 may vary depending on cell line and conditions, but ChIP-Seq analysis from public data sets showed binding of TCF7L2 in a number of human cell lines at Atp2a2 (Supplemental Figure 5I). Motif interrogation identified multiple potential binding sites of TCF7L2 near the *Lexis* binding site at the Atp2a2 promoter region. To confirm TCF7L2 binding at Atp2a2, we performed ChIP-qPCR in WT and TCF7L2 KO preadipocytes (64). We confirmed binding of TCF7L2 at the endogenous promoter of Atp2a2, and no binding was observed in TCF7L2-deficient preadipocytes (Figure 5J). As a positive control, we also confirmed binding of TCF7L2 at the canonical WNT target *Axin2* (Supplemental Figure 5J). In addition, we found that TCF7L2 bound the Atp2a2 gene region in iWAT and, interestingly, its binding was modestly but significantly enhanced in *Lexis*-KO cells (Figure 5J). Complementing this approach, electromobility shift assays confirmed TCF7L2-Atp2a2 DNA interaction. The use of a competitive inhibitor abolished the TCF7L2 binding to the ATP2a2 probe, indicating that the binding of TCF7L2 to Atp2a2 is sequence dependent. TCF7L2 antibody further increased the mobility shift of the TCF7L2-Atp2a2 complex (super shift), suggesting that the binding of the *Atp2a2* probe involves TCF7L2 (Supplemental Figure 5K). Collectively, these results indicate the *Lexis* is enriched at TCF7L2 binding sites and hint that *Lexis* may modulate the activity of TCF7L2 at target genes including Atp2a2.

### Effects of Lexis on gene expression are dependent on TCF7L2.

**Figure 6:**
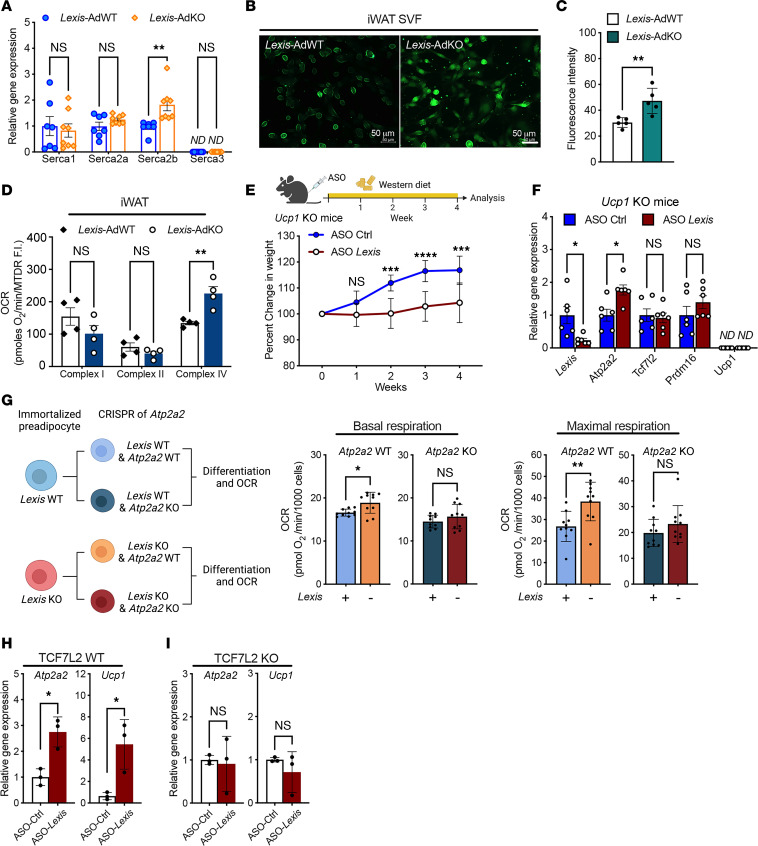
TCF7L2 is required for the effects of *Lexis* on thermogenesis. (**A**) Relative mRNA levels of Serca1, Serca2 (Serca2a and Serca2b, coded by Atp2a2), and Serca3 in iWAT of *Lexis*-AdWT or *Lexis*-AdKO mice 12-week WD-fed detected by qRT-PCR (*n* = 7 for *Lexis*-AdWT; *n* = 8 for *Lexis*-AdKO). (**B**) Images of fluorescent intracellular Ca^2+^ levels in SVF from *Lexis*-AdWT or *Lexis*-AdKO mice with NE stimulation. (**C**) Fluorescence intensity quantification of **B** (*n* = 5 per group). (**D**) Average OCR using iWAT from *Lexis*-AdWT or *Lexis*-AdKO mice (*n* = 4 per group). OCR was normalized to mitochondrial content. (**E**) Ucp1-KO male mice (10–12 weeks) treated with ASO Ctrl or ASO *Lexis* (*n* = 6 per group). (**F**) Gene expression by qRT-PCR from iWAT of mice from **E**. (**G**) Schematic for generation of *Atp2a2*-KO preadipocytes. Preadipocytes in each condition were induced by browning cocktail for 5 days and OCR was detected (*n* = 10 per group). Basal and maximal respiration shown. (**H**) Gene expression of Atp2a2 and Ucp1 detected by qRT-PCR from TCF7L2 WT preadipocytes treated with ASO Ctrl or ASO *Lexis* (*n* = 3 per group). (**I**) Gene expression of Atp2a2 and Ucp1 detected by qRT-PCR from TCF7L2-KO treated with ASO-Ctrl or ASO-*Lexis* (*n* = 3 per group). Data are represented as mean ± SEM (**A**, **D**, and **F**) or mean ± SD (**C**, **E**, **G**, **H**, and **I**). *P* values were calculated by either unpaired *t* test (**A**, **C**, **D**, **F**–**I**) or 2-way ANOVA (**E**). *P < 0.05; **P < 0.01; ***P < 0.001; ****P < 0.0001.

To better understand the functional relationship among *Lexis*, TCF7L2, and Atp2a2, we probed for SERCA2 alterations as a consequence of loss of *Lexis*. In vivo, adipose-specific loss of *Lexis* enhanced SERCA2 expression detected by qPCR in iWAT from WD-fed mice, while the expression of other SERCAs was not significantly altered (Figure 6A). In addition, we treated *Lexis*-AdKO mice and *Lexis*-AdWT mice with the PPARγ agonist rosiglitazone for 7 days (Supplemental Figure 6B) (66) and noted an increase in *Ucp1*, *Cox8b*, and *Serca2 in Lexis*-AdKO (although Serca2 was not significant) in iWAT without an increase in TCF7L2 or PRDM16 (Supplemental Figure 6A). Although the trend was for increased expression, not all genes bound by *Lexis* showed significant changes in gene expression in the *Lexis*-KO (Supplemental Figure 6B).

Increased SERCA2 is associated with enhanced Ca^2+^ cycling (1, 23). We therefore compared the intracellular Ca^2+^ level in SVF from iWAT of *Lexis*-AdKO mice and *Lexis*-AdWT mice with norepinephrine (NE) treatment. Cell Ca^2+^ staining showed higher intracellular Ca^2+^ levels after NE stimulation in SVFs from *Lexis*-AdKO mice compared with *Lexis*-AdWT mice (Figure 6, B and C). In agreement with these results, intracellular Ca^2+^ concentration measurement by detecting fluorescence activity showed moderate but significantly higher NE-stimulated intracellular Ca^2+^ in *Lexis*-AdKO SVFs (Supplemental Figure 6B). Consistent with the increase in UCP1 and calcium cycling, differentiated SVF induced by browning cocktail from iWAT of *Lexis*-AdWT mice or *Lexis*-AdKO mice showed an increase in maximal respiration (Figure 6D). Taken together, these data suggest that effects of *Lexis* on energy expenditure and body weight may not be exclusively dependent on UCP1. To directly underpin the contributions of UCP1-independent heat dissipation, we treated *Ucp1*-KO mice with control or *Lexis* ASO. *Lexis* ASO–treated mice had lower weight gain compared with controls and showed enhanced *Atp2a2* (Figure 6, E and F). Notably, genetic deletion or pharmacological inhibition of Atp2a2 by thasigargin diminished the oxygen consumption rate (OCR) difference imparted by loss of *Lexis* (Figure 6G and Supplemental Figure 6, D and E), suggesting at least some of the effects of *Lexis* are dependent on *Atp2a2*. Collectively, these results also indicate that the *Lexis* effects on adiposity do not hinge on changes in UCP1.

To test the dependency of *Lexis* effects on TCF7L2, we treated control or TCF7l2 null adipocytes (Supplemental Figure 6, F and G) with *Lexis* ASO or control ASO. In line with other results, both Serca2 and Ucp1 were higher due to loss of *Lexis* in control adipocytes (Figure 6, H and I). However, the gene expression changes were abrogated in TCF7L2 null cells (Figure 6, H and I, and Supplemental Figure 6, H–J), suggesting the regulatory effect of *Lexis* on SERCA2, UCP1, and other thermogenic genes were dependent on TCF7L2 in adipocytes. It should be noted that not all genes regulated by *Lexis* in vivo were highly sensitive to loss of TCF7L2 in the SVF model (example, *Cidea;* Supplemental Figure 6I). Taken together, these results suggest that the effect of *Lexis* on thermogenic gene expression requires TCF7L2.

## Discussion

In this paper, we identify a diet and thermoresponsive lncRNA as a critical determinant of WAT energy expenditure. Adipose tissue thermogenesis is tightly orchestrated by coordinate transcriptional events that couple environmental cues to bioenergetic changes (4). Cold exposure, exercise, and cachexia activate beiging in white adipose depots through the activity of panadipocyte factors such as PPARγ and beige-specific mediators such as PRDM16 (4). Although much is known about synchronized control mechanisms that activate beiging, little is known about how beige adipocytes are “turned off” or impoverished during thermoneutral exposure or DIO. The identification of a PPARγ/*Lexis* axis fills fundamental gaps on this front. Loss of *Lexis* had no effect on thermogenesis during cold exposure, perhaps due to the almost nondetectable levels of *Lexis* during cold. However, calorie-rich feeding or thermoneutral exposure activates PPARγ and, in turn, *Lexis,* which suppresses beige adipocyte enrichment. It is conceivable that this adaptive mechanism exists to minimize energy utilization and prioritize fat storage. PPARγ, like other type II nuclear receptors, induces gene expression in response to endogenous mediators (fatty acids) or pharmacologic stimulation, but despite direct binding of PPARγ at thermogenic gene promoters, their expression is only induced by PPARγ under certain conditions, even when endogenous receptor ligands are highly enriched. The discovery of a role for *Lexis* in modulating thermogenic genes offers insights as to how nuclear receptors can dynamically fine-tune transcriptional outputs and expands the metabolic contributions of noncoding gene regulatory circuits.

lncRNAs have emerged as important mediators in biology, with many functioning at the interface of chromatin and the genome. However, we lack mechanistic understanding for the vast majority of functional lncRNA effects, since RNA-centric interrogation approaches, although invaluable, can be tedious and require substantial optimization (32). For example, techniques such as ChIRP and RNA antisense purification (RAP) provided crucial insights into the molecular activities of *Xist*, arguably the best-characterized lncRNA, but have been rarely used to study metabolism (33, 34). Here, we used similar unbiased chromatin affinity assays to map the binding sites and motif preferences of *Lexis*. Aligned with our functional studies, we find that *Lexis* bound genes involved in canonical thermogenesis, but unexpectedly, *Lexis* also directly targeted ATP2a2, a UCP1-independent mediator of heat dissipation, by regulating calcium flux (20, 23). These results imply that canonical and noncanonical thermogenesis are coordinately integrated within white adipose depots. In addition, we found that *Lexis* is preferentially enriched at binding sites of the WNT transcriptional mediator TCF7L2. *Lexis* effects were dependent on TCF7L2, and interestingly, mice lacking TCF7L2 in adipose tissue showed a phenotype opposite that of the *Lexis* KO (64). It is known that polymorphisms at TCF7L2 are strongly associated with risk of diabetes, obesity, and other metabolic disturbances, hinting that our results may be relevant for human disease. Furthermore, our findings expand the repertoire of opposing interactions between PPARγ and WNT/β-catenin in shaping adipose fate (63). It is known that WNT activation inhibits adipogenesis and its tight regulation is important for proper organismal development (62); however, the role and mechanisms of WNT signaling in mature adult adipose depots have proven to be more difficult to elucidate. Notably, temporospatial control of WNT activity in specific adipose populations would be challenging to accomplish, and it is often difficult to disentangle cell-autonomous versus secondary effects when disrupting endogenous WNT regulators in KO models. Our work here circumvents some of these challenges, suggesting that TCF7L2 plays a direct role in thermogenic gene regulation.

The effort to understand the cellular origins of beige cells under conditions where they are highly enriched, such as cold exposure or excessive β-adrenergic signaling, has attracted substantial attention (1, 67), but multiple lines of evidence suggest that adipose thermal homeostasis may be physiologically relevant even in the absence of such signals. For example, knockout of PRDM16 in beige adipocytes in a DIO model is associated with increased weight and metabolic disturbances (18). In addition, a number of groups have identified regulated cell populations in mouse iWAT expressing thermogenic markers at room temperature (68, 69). This current study expands the role of “homeostatic” thermoregulation, highlighting the existence of unique regulatory circuits that temper beige cell enrichment in response to HFD feeding and thermoneutrality. Our single-cell analysis identified a number of populations expressing thermogenic markers, the majority of which did not differ between WT and KO and that had overlapping markers with other cell types, such as mature adipocytes. These results are in line with a recent single-cell atlas analysis showing that a clearly delineated murine thermogenic population in WT iWAT may be difficult to identify, especially during calorie-rich feeding (68). However, the most dominant change in our analysis is the almost exclusive enrichment in the *Lexis* AdKO of C6 cells expressing thermogenic markers. Beige adipocytes are unlikely to be uniform in origin and may be enriched in adipose depots through distinct mechanisms, including de novo differentiation of more than one progenitor cell type, conversion from mature adipocytes, or proliferation of progenitors or mature cells (3). Aligned with our results, a study published at the time of this writing used a β-catenin reporter system to show that a distinct population of beige adipocytes is driven by enhanced WNT signaling (70). It is also worth noting that a recent study tracing the origin of cold-induced beige fat suggests that the proliferative capacity of some APCs is a critical determinant of beiging (67). The same work also hints that certain populations in iWAT may possess intrinsic thermogenic activity even in the absence of adrenergic stimulation (67). Thus, it is tempting to speculate that “innate” thermogenesis in iWAT may be disease relevant and, despite some overlap, employs unique regulatory mechanisms compared with adaptive thermogenic responses in the same adipose depot. Future work will expand on the mechanisms involved in regulation of thermogenic populations in response to specific environmental cues.

The significance of lncRNAs is occasionally questioned owing to their subtle effects in KO models or in vivo perturbations (71, 72), but our work suggests that context is critical to interpretation. Loss of *Lexis* under cold exposure, the canonical condition that stimulates beiging, resulted in no major phenotypic differences. These results stand in contrast to differences between WT and KO under thermoneutral conditions and dietary stress. Intriguingly, different isoforms of *Lexis* dominate in adipose tissue versus liver and future work will focus on the biochemical and structural basis for *Lexis* function as well as deciphering different domains that may be required for its activity. Our previous work showed that *Lexis* directly interacts with RALY and antagonizes its broad transcriptional coactivator properties (29, 73). Interestingly, lethal yellow, a classic mouse model of obesity and insulin resistance, is characterized by a spontaneous mutation that leads to deletion of RALY and overexpression of the neighboring agouti gene (74). Although the dominant ectopic of agouti is thought to be the sole contributor to obesity in lethal yellow (75), based on this work, we ponder whether loss of *Raly* may at least in part contribute to the metabolic disturbances in lethal yellow. Notably, variants in RALY are associated with cardiometabolic abnormalities (76, 77). Finally, this work provides evidence that a nontissue selective *Lexis* ASO treatment offers partial protection from weight gain in a DIO model, forecasting that specific inhibition of *Lexis* in adipose depots may be beneficial in metabolic diseases.

## Methods

Detailed methods are provided in Supplemental Methods. See complete unedited blots in the supplemental material. All the materials including antibodies, reagents, kits, enzymes, inhibitors, mice, and cell lines as well as their recourses are included in Supplemental Table 2.

### Cell culture.

All preadipocytes were cultured in DMEM with 10% FBS. The TCF7L2 WT and TCF7L2 KO preadipocytes were generated as previously described (64). Preadipocytes were differentiated according to previous protocols with minor modifications (78, 79). Adipose-derived mesenchymal stem cells (PCS-500-011) were purchased from ATCC and cultured and differentiated according to ATCC protocol.

### Animals.

All mice used in the study were on a C57BL/6 background. Most experiments used mice approximately 10 to 12 weeks old. Mice were fed chow diet, WD, or HFD (Research Diet) and housed in a temperature-controlled room under a 12-hour light/12-hour dark cycle and pathogen-free conditions. *Lexis* global KO mice and TCF7L2^fl^ mice were generated per our previous study (29, 64). *Lexis^fl^* mice were generated by Cyagen Biotechnology. Adipose tissue–specific KO mice and littermate controls were generated by crossing *Lexis^fl^* with Adipoq-cre mice (Jackson Laboratory) as a strategy outlined in Supplemental Figure 3A. *Ucp1*-KO mice were bought from Jackson Laboratory. Experiments used male or female mice as indicated, except thermoneutrality studies included a mix of both. *Ucp1*-KO mouse experiments were pooled from 2 smaller studies, since we were not able to generate a sufficient number of *Ucp1*-KO in a single cohort.

### Single-nucleus RNA-Seq.

Nuclei of iWAT were isolated from WD-fed male mice using a protocol slightly modified from recent publications (38, 39). We performed 10× Genomics single-nucleus RNA-Seq at the USC Norris Molecular Genomics Core (Los Angeles, California, USA) immediately after nuclei isolation.

### Chromatin-affinity assays.

ChIRP qPCR in preadipocytes was performed according to previous publication (46). A total of 50 million GW1929-treated preadipocytes (24 hours) were used for each replicate. The sequences of probes and primers are provided in Supplemental Table 3. The HiChIRP assay was performed according to the published protocol with modifications (30). A total of 100 million GW1929-treated preadipocytes (24 hours) were used for each replicate, and 2 replicates were performed for each group. As negative control, RNase A and RNase H were added (2 μg per million cells). Libraries were paired end sequenced by NovaSeq S4 with read lengths of 150 bp at the Broad Stem Cell Research Center (BSCRC) Sequencing Core at UCLA. For the data analysis, raw reads were uniquely mapped to reference mouse genome (mm9) using Bowtie2 (80). MACS2 was used for peak calling (81, 82). Motif analysis was performed using *Lexis* genomic binding sites and visualized by PscanChIP and MotifStack (83, 84).

### Statistics.

Unless specified, a 2-tailed, nonpaired Student’s *t* test or ANOVA with multiple comparisons (more than 2 experimental groups) was used to determine statistical significance. A *P* value of less than 0.05 was considered significant. Data are represented as mean ± SD or mean ± SEM, as specified in figure legends. Group sizes were based on statistical ANOVA and prior experience with similar in vivo studies. For analysis of energy expenditure, data were generated by the indirect calorimetry experiments as previously described (85), using the method provided by the National Institute of Diabetes and Digestive and Kidney Diseases (NIDDK) Mouse Metabolic Phenotyping Centers using their Energy Expenditure Analysis page (http://www.mmpc.org/shared/regression.aspx) or CalR 1.3 (86).

### Study approval.

All experiments were approved by the UCLA Institutional Animal Care and Research Advisory Committee and performed in accordance with the recommendations in the *Guide for the Care and Use of Laboratory Animals* (National Academies Press, 2011).

### Data availability.

All the deep sequencing data generated in this paper have been submitted to the NCBI’s Gene Expression Omnibus database (GEO GSE215870, GSE216272, and GSE216608). Values for all data points in graphs are reported in the Supporting Data Values file.

## Author contributions

TS conceptualized the project. TS and ZZ designed the research. TS secured funding for the project. ZZ, VS, DW, MJT, XW, JK, PR, and SZ performed the research and analyzed data. YC, LC, ZZ, and WL performed the computational analysis. ZZ, MJT, PR, PT, and CJV provided valuable resources and assisted with results interpretation. ZZ and TS drafted the manuscript. All authors participated in the editing process, brought intellectual input, and approved the manuscript.

## Supplementary Material

Supplemental data

Supporting data values

## Version 1 11/01/2023

Electronic publication
