# Supplementary material for: A PPARγ/long noncoding RNA axis regulates adipose thermoneutral remodeling in mice
Source: J Clin Invest. 2023 Nov 1;133(21):e170072. doi: 10.1172/JCI170072 (PMC10617768; doi:10.1172/JCI170072)
Supplement: Supplemental data [file jci-133-170072-s034.pdf]

Supplemental Material

A PPARγ-long noncoding RNA axis regulates adipose thermoneutral remodeling in mice

Table of Contents

Supplemental Methods **Page 2**

Supplemental Figures **Page 12**

Supplemental Figure 1 *Page 12*

Supplemental Figure 2 *Page 14*

Supplemental Figure 3 *Page 16*

Supplemental Figure 4 *Page 18*

Supplemental Figure 5 *Page 19*

Supplemental Figure 6 *Page 21*

Supplemental Tables **Page 23**

Supplemental Table 2 *Page 23*

Supplemental Table 3 *Page 24*

Supplemental Table 3 *Page 26*

References **Page 27**

Supplemental Methods

Cell culture

C3H/10T1/2 (10T1/2), 3T3L1, TCF7L2 WT and TCF7L2 KO preadipocytes were cultured in DMEM with 10% FBS. The TCF7L2 WT and TCF7L2 KO preadipocytes were generated as previously described (1). Preadipocytes were differentiated according to previous protocols with minor modifications (2, 3). Briefly, for whitening cocktail treatment, 1 million cells were plated in 6-well plate grown overnight or until confluence, then induced by 2 ml differentiation cocktail (Insulin: 5 µg/ ml; dexamethasone: 0.5 µM; GW1929: 50 nM; IBMX: 0.5 mM) for 2 days, followed by adding maintenance media (Insulin: 5 µg/ ml). For browning cocktail treatment, 2 ml browning cocktail (Insulin: 5 µg/ml; dexamethasone: 0.5 µM; IBMX: 0.5 mM; T3: 1 nM; Indomethacin: 0.125 mM; GW1929: 50 nM or rosiglitazone: 1 uM) was added and changed to maintenance media (Insulin: 5 µg/ ml, T3: 1nM, GW1929: 50 nM or rosiglitazone: 1 uM) after two days.

Adipose-Derived Mesenchymal Stem Cells (ADMSC, PCS-500-011) were purchased from ATCC. For cell culture, Mesenchymal Stem Cell Basal Medium (ATCC, PCS-500-030) plus one Mesenchymal Stem Cell Growth Kit (ATCC, PCS-500-040) were used according to ATCC recommended protocol. Cells were split and grown to 70-80% confluency using Trypsin-EDTA for Primary Cells (ATCC, PCS-999-003) and the Trypsin Neutralizing Solution (ATCC, PCS-999-004). To induce adipocyte differentiation, cells were cultured in 6-well plate at 37°C with 5% CO_2 for 48 hours before differentiation with 2 ml/ well of Adipocyte Differentiation Initiation Medium containing Adipocyte Basal Medium and AD Supplement at a ratio of 15:1. Cells were incubated at 37°C with 5% CO2 for 48 hours followed by 2 ml/ well of pre-warmed Adipocyte Differentiation Maintenance Medium containing Adipocyte Basal Medium and ADM Supplements (supplied in Mesenchymal Stem Cell Growth Kit) at the ratio of 17:1. RNA was collected in Trizol at D0, D8 and D12 post induction.

Animals

All mice used in the study were in C57BL/6 background. Most experiments used ~10-12 weeks old mice. Mice were fed chow diet, western or high-fat diet (Research Diet) and housed temperature-controlled room under a 12-h light/12-h dark cycle and pathogen-

free conditions. *Lexis* global knockout mice and TCF7L2^{flox} mice were generated per our previous study (4) (1). *Lexis*^{flox} mice were generated by Cyagen Biotechnology using gene-targeting technology. Adipose tissue specific knockout mice and littermate controls were generated by crossing with Adipoq-cre mice (Jackson Laboratory) using the strategy outlined in Supplemental Figure 3 (A) were created in our previous study *Ucp1* KO mice were bought from Jackson Laboratory (003124, Jackson Laboratory). Experiments used male or female mice as indicated except thermoneutrality studies included a mix of both. *Ucp1* KO mice experiments were pooled from two smaller studies since we were not able to generate a sufficient number of *Ucp1* KO in a single cohort.

Anti-sense oligonucleotide (ASO) studies

Acute *Lexis* antisense oligonucleotide (ASO) studies were performed as we previously described (2, 4). Male mice at 9-10 weeks of age were intraperitoneally injected with control or *Lexis* ASO at the dose of 25 mg /kg twice a week. Body weight was measured weekly and body composition was determined by EchoMRI Body Composition Analyzer.

Diet-induced Obesity Studies

The majority of studies used mice aged 8-12 weeks old and fed a calorie-rich diet (Western Diet, D12079B, Research Diet). Although in pilot studies we tested Western and High Fat diet, we used Western Diet for most experiments because the induction of *Lexis* was noted to be highest. Body composition was determined by EchoMRI analysis (EchoMRI, 3-in-1). For the food intake experiment, mice were singly housed in standard housing and fed chow or western diets. The mass of diet administered and consumed was recorded daily over a period of seven days and shown as gram of diet per mouse per day.

Thermal stress challenge

For the thermoneutrality studies, mice were group housed in monitored climate-controlled facilities maintained on a 12 h light–dark cycle at a constant temperature of 30 °C. For chronic cold exposure studies, mice were group housed and placed at 6°C with bedding and free access to food and water. The rectal temperature of mice was monitored using Bioseb rodent thermometer (BIOSEB)

Metabolic chamber studies

Energy expenditure was measured using male mice by indirect calorimetry with chow or Western Diet feeding as indicated in figure legend (Oxymax CLAMS, Columbus Instruments). We detected the body composition and body mass right before the calorimetry experiments. The indirect calorimetry experiment generated data was analyzed using CalR (5).

Glucose tolerance tests (GTT) and insulin tolerance tests (ITT)

GTT and ITT were performed using our previous described method (2). Briefly, for glucose tolerance tests, we fasted the mice for 6 hours and then challenged them with an IP injection of glucose with the amount of 1 g kg^{-1} (according to lean mass or total body mass as indicated in figure legends). For insulin tolerance tests, we fasted the mice for 6 hours and i.p. injected insulin with the amount of 1 U kg^{-1}. Blood glucose levels were detected at different time points as shown in the figures using the ACCUCHEK active glucometer (Roche).

Cellular and Mitochondrial Respiration assay

Tissue OCR was measured through the Mitochondrial and Metabolism Core at UCLA using mitochondrial isolated from iWAT samples according to previous publications with modification (6, 7). Briefly, frozen iWAT samples were stored in -80 °C until use in the Seahorse experiments. Frozen tissues were thawed on ice and homogenized in 1 mL MAS buffer (70 mM sucrose, 220 mM mannitol, 5 mM KH2PO4, 5 mM MgCl2, 1 mM EGTA, 2 mM HEPES pH 7.2) with 15-20 strokes in a Dounce tissue homogenizer. Homogenates were centrifuged at 20,000 × g for 10 minutes at 4 °C, the supernatant was removed, and the pellet was resuspended in 75 µL MAS buffer. The sample was then centrifuged at 500x g and the supernatant was collected. Protein concentrations were determined by BCA assay kit (Thermo Fisher). Homogenates were loaded into Seahorse XF96 microplate at 4 µg/ well in MAS buffer (20 µL each well) and centrifuged at 2,000× g for 5 minutes at 4 °C. After centrifugation the volume was increased to 150 µL by adding 130 µL MAS containing cytochrome c (10 µg/mL). Substrate injections at port A included final concentrations of 1 mM NADH to determine the respiratory capacity of mitochondrial Complex I or 5 mM succinate with 2 µM rotenone to determine the respiratory capacity of mitochondrial Complex II. The following compounds were

injected sequentially to final concentration of 2 μM rotenone with 4 μM antimycin A (Port B); 0.5 mM TMPD with 1 mM ascorbic acid (Port C); and 50 mM azide (Port D). OCR rates were measured using Seahorse XF96 Extracellular Flux Analyzer (Agilent Technologies) and normalized to mitochondrial content quantified by MitoTracker Deep Red (MTDR). Homogenates were stained with 500 nM MTDR for 10 minutes followed by two wash steps to remove the dye (Thermo Fisher). MTDR fluorescence was read on a Tecan Spark plate readers (Ex: 633 nm; Em: 678 nm).

For oxygen consumption rate (OCR) detection in adipocytes differentiated from SVF, 40,000 beige adipocytes were plated in each well of a XF24-well cell culture plate in 100 μL of DMEM culture media. After 2 hours attachment, another 400 μL DMEM culture media was added and allowed to attach overnight. The culture media was replaced the next day with MAS buffer (70 mM sucrose, 220 mM mannitol, 10 mM KHPO4, 5 mM MgCl2, 2 mM HEPES, 1.0 mM EGTA and 0.2 % fatty acid-free BSA) containing 40 μM palmitoyl-carnitnine and 500 μM malate. Plasma membrane was permeabilized with Plasma Membrane Permeabilizer reagent (Agilent) according to the manufacturer protocol. OCR was measured before and after the sequential injections of 2.5 μM oligomycin, 4 μM FCCP, and 4 μM antimycin A for the coupling assay, and 2 μM rotenone, 10 mM succinate, 4 μM antimycin A, and 100 μM TMPD for the electron flow assay, using an XF24 Seahorse Bioscience instrument. UCP1-dependent respiration was assessed by injecting 1 mM GDP and represents the GDP-sensitive respiration. All OCRs were normalized by protein using a Bradford assay after respirometry measures.

OCR detection using immortalized preadipocytes in CRISPR KO or thapsigargin treatment experiments was performed through the Mitochondrial and Metabolism Core at UCLA. Briefly, 15,000 preadipocytes were placed in each well of Seahorse XF96 microplate, induced by browning cocktail for 5 days and then measured using an XF96 Extracellular Flux Analyzer (Seahorse Biosciences). OCRs were normalized to cell numbers.

Gene expression analysis and immunoblot analysis

TRIzol reagent (Invitrogen) was used for total RNA isolation. Random Hexamer Primers and Olig-dT primers were used for reverse transcription. Gene expression was quantified using cDNA by qRT-PCR using SYBR Green Master Mix (Bio-Rad) on Bio-

Rad 384 Real-time PCR instrument. Gene expression levels were determined by using a standard curve or 2(-Delta-Delta C(T)) method. Each gene was normalized to the housekeeping gene 36B4. For immunoblot analysis, whole cell lysate or tissue lysate was extracted using RIPA lysis buffer (Boston Bioproducts) supplemented with complete protease inhibitor cocktail (Roche). Proteins were diluted in Nupage loading dye (Invitrogen), heated at 95 °C for 5 min, and run on 4–12% NuPAGE Bis-Tris Gel (Invitrogen). Proteins were transferred to PVDF blotting membrane (0.45 μm) blocked with 5% milk to quench nonspecific protein binding and blotted with the indicated primary antibody. For complete listing of antibodies please see Supplemental Table 2.

Haematoxylin & Eosin (H&E) staining

All paraffin embedment and H&E staining in this paper was performed by the Translational Pathology Core Laboratory (TPCL) in UCLA. The iWAT tissues of mice were fixed in 10% formaldehyde overnight, washed with tap water for 15 minutes and stored in 70% ethanol before being mounted in paraffin. After Paraffin embedment, 5 μm sections were sliced and stained with haematoxylin and eosin. Images were taken with a fluorescence microscope (ZEISS).

Subcellular fractionation

A total of 20 million preadipocytes were fractionated into cytosolic and nuclear compartments using detergent lysis according to published protocol with modification (8). Briefly, after collection and washing with cold PBS, cells were resuspended in cold cytoplasmic buffer (0.15% NP-40, 10 mM Tris pH 7.5 and 150 mM NaCl) and incubated on ice for 10 min. Lysates were layered onto 2.5 volumes of a chilled sucrose buffer (10 mM Tris 7.5, 150 mM NaCl, 24% sucrose) and spined at 14,000 rpm× 10 min× 4 °C. Supernatant was kept as cytoplasmic lysate. Nuclei were resuspended with glycerol buffer (20 mM Tris pH 7.9, 75 mM NaCl, 0.5 mM EDTA, 50% Glycerol and 0.85 mM DTT), then incubated by cold nuclei lysis buffer (20 mM HEPES-pH 7.6, 7.5 mM $MgCl_2$, 0.2m M EDTA, 0.3 M NaCl, 1 M Urea, 1% NP-40 and 1 mM DTT) on ice for 10 min and spined at 14,000 rpm× 2 min× 4 °C. Supernatant was kept as nuclei lysate.

Stromal vascular fraction (SVF) isolation and immortalization

SVFs were isolated from the iWAT of 4-6 weeks old mice and induced for differentiation based on previous published protocol (1, 9). SVF were immortalized by retroviral

expression of the SV40 large T antigen according to our previous publication (10). Genetic deletion by the CRISPR-Cas9 system or pharmacological inhibition of *Atp2a2* by thaipsigargin (2 μM) as previously described (11). We used the exact guide RNA sequences published by Ikeda et al., (11). To generate lentivirus vector, 1,200 ng Lenti-CRISPR v2 expressing guide RNA (or Control) and Cas9 were co-transfected with Rev (360 ng), pMDL (780 ng) and VSV-G (420 ng) into 1 million HEK293T cells in each one well of a 6-well plate. 48 hours post transfection, supernatants containing lentivirus were filtered by 0.45 μm Millex®-HV Sterile Filter (Millipore) and used for preadipocytes infection. After infection and puromycin selection, preadipocytes were plated in Seahorse XF96 microplate with amount of 15,000 cells per well, induced for differentiation by browning cocktail and OCRs were detected as described above.

Intracellular calcium assay

Intracellular calcium assays were performed from Fluo-8 Calcium Flux Assay Kit - No Wash (Abcam) according to previous publication with minor modifications (11). Briefly, SVF derived from iWAT of *Lexis*-AdWT or *Lexis*-AdWKO mice were plated in 96-well plates and followed by overnight culture. After incubating with Fluo-8 for 1 hr at RT in calcium-free Hanks' balanced salt solution, fluorescence intensity was quantified at Ex/Em = 490/525 nm. Intracellular calcium staining in SVF was performed using Fluo-8 AM, green-fluorescent calcium binding dye (Abcam, ab142773). Briefly, cells were seeded in cell culture chamber slide (Nest Scientific) at low density. Calcium indicator Fluo-8 AM was loaded at a concentration of 4 uM for 1 hour in Hanks Balanced Salt Solution with 20 mM HEPES (HHBS buffer) in 37 °C with 5% CO_2. Excess fluo-8 was removed by washing twice with 200 ul HHBS buffer. To stimulate Ca^{2+} transients, cells were treated by HHBS buffer containing 1 uM NE for 2 min. The stained cells were imaged with a fluorescence microscope (ZEISS) using an FITC channel. Fluorescence intensity was quantified by ImageJ software.

RNA-seq and data processing

RNA-seq was performed in Technology Center for Genomics & Bioinformatics (TCGB) at UCLA. Libraries for RNA-Seq were prepared with KAPA Stranded RNA-Seq Kit on RNA isolated from iWAT of 12 weeks western diet fed *Lexis*-AdWT or *Lexis*-AdKO mice. The data were sequenced on Illumina HiSeq 3000. RNA-seq reads were aligned with

HISAT2 to the mouse genome (mm9) (12). Differential gene expression analysis was performed using R package DEseq2 (13). Enrichment analysis for Gene Ontology terms among the differential expressed genes was performed using Metascape (14). For analysis of RNA-seq data download from public dataset under GEO number GSE94654 (2) or GSE151324 (15), reads were aligned to mouse genome (mm39) or human genome (GRCh38) with HISAT2 (12). FeatureCounts (16) was applied to call read counts of lncRNAs for each sample with the mice gene annotation of GENCODE (Release M31, GRCm39) or human gene annotation of GENCODE (Release 42, GRCh38.p13) (17). Differential gene expression analysis was performed using R package DEseq2 (13).

Chromatin-affinity assays

Chromatin Isolation by RNA Purification (ChIRP) qPCR in preadipocytes was performed according to previous published protocol (18). Cells were treated with GW1929 for 24 hours before ChIRP steps. A total number of 50 million preadipocytes were used for each replicate and 3 replicates were performed for each group. Cells are crosslinked in 1% fresh made Glutaraldehyde for 12 min. After cell lysis, sonication was performed using Covaris E220 according to the manufacturer's protocol. The sequence of primers used for ChIRP-qPCR were provided in Supplemental Table 3. HiChIRP assay was performed according to the published protocol with modification (19). Briefly, a total of 100 million preadipocytes (pre-treated with GW1929 for 24 hours) were used for each replicate and 2 replicates were performed for each group. For negative control, RNase A and RNase H were added as the amount of 2 µg per million cells. Covaris E220 was used for sonication with the following parameters: Fill Level = 10, Duty Cycle = 5, PIP = 140, Cycles/Burst = 200, Time = 45 min. For sequencing, libraries were paired-end sequenced by NovaSeq S4 with read lengths of 150 bp at Broad Stem Cell Research Center (BSCRC) Sequencing Core at UCLA. For the data analysis, raw reads were uniquely mapped to reference mouse genome (NCBI37/mm9) using Bowtie2 (20). MACS2 was used for peak calling (21, 22). Motif analysis was performed based on *Lexis* genomic binding sites and visualized by PscanChIP and MotifStack (23, 24).

***Lexis* binding sites prediction**

Software "LongTarget" was used to predict DNA binding motifs and binding sites of *Lexis* in a genomic region based on potential base pairing rules between an RNA sequence and a DNA duplex according to published methods (25, 26).

Chromatin Immunoprecipitation (ChIP)

ChIP was performed following previously published protocols with minor modifications (2, 27, 28). For ChIP using preadipocytes, 20 million cells were lysed and sonicated using M220 Focused-ultrasonicator (Covaris) according to the manufacturer's protocol. Chromatin was immunoprecipitated with 5 µg antibodies against TCF7L2 antibody (Cell Signaling Technology) or IgG (Millipore) overnight at 4°C. For iWAT ChIP, adipocyte nuclei isolation from 500 mg of iWAT from 10-week-old *Lexis*-AdWT or *Lexis*-AdKO mice were performed as previously described (9). After reversal of cross-linking, DNA was isolated and DNA enrichment was quantified by qRT-PCR. *Axin2* was used as positive control of TCF7L2 ChIP-qPCR according to previous publication (29). The sequence of primers used for ChIP-qPCR is provided in Supplemental Table 3.

RNA fluorescence in situ hybridization (RNA FISH)

Custom design RNA FISH probes against *Lexis* were designed using Stellaris FISH probe designer and ordered from Stellaris (LGC Biosearch Technologies). Sequences of RNA FISH Probes were provided in Supplemental Table 3. Probes were labeled with Quasar® 570 Dye and single molecule RNA FISH was performed according to Stellaris® RNA FISH protocol. For nuclei staining, slides were mounted with ProLong Diamond Antifade Mountant with DAPI (ThermoFisher Scientific). Fluorescent signals were captured using a laser scanning confocal microscope (SP80, Leica).

Electrophoretic Mobility Shifting Assay (EMSA)

EMSA was performed to determine the binding of TCF7L2 to the Atp2a2 promoter region bearing one putative TCF7L2 recognition site (Sequences in Supplemental Table 3) in preadipocyte. The corresponding 3' end biotin labeled oligonucleotides were synthesized by Integrated DNA Technology (IDT). Nuclear extracts were prepared using the NE-PER Nuclear Extraction Reagent Kit (ThermoFisher Scientific). The LightShift EMSA Kit (ThermoFisher Scientific) was used for binding reaction according to the manufacturer's profocol. 20 fM concentration of labeled duplex DNA and 4 mg of nuclear protein extract were used in the reaction. For super-shift, we added 1 µl of

TCF7L2-specific ChIP grade antibody (Cell Signaling Technology) to nuclear extract proteins for 30 min at room temperature before adding labeled probes. The electrophoresis was performed by running reaction mix on 5% native agarose gel in 0.5X TBE buffer. After transfer onto a positively charged nylon membrane (ThermoFisher Scientific), DNA-protein complex was crosslinked by UV and visualized with Horseradish Peroxidase-Conjugated Streptavidin (ThermoFisher Scientific).

Single-nucleus RNA-seq

Isolation of nuclei from iWAT of western diet fed mice

We isolated the nuclei of iWAT from western diet fed male mice using a protocol slightly modified from recent publications (30, 31). Briefly, iWAT from 3 mice of *Lexis*-AdWT group or *Lexis*-AdWT group were isolated (without lymph node) pooled (according to group) and minced. 2 ml NIB buffer (250 mM Sucrose, 10 mM HEPES, 1.5 mM MgCl2, 10 mM KCl, 0.001% IGEPAL CA-630, 0.2 mM DTT, 0.5 U/µL RNase inhibitor) were added to 400 mg pooled iWAT, then homogenized with 2 ml glass dounce homogenizer. After filtering through 70 µm cell strainer, another 2 ml NIB was added to the filtered homogenate and centrifuged at 1000 g× 10 min× 4 °C. The lipid layer was aspirated, pellet was resuspended in the remaining supernatant and then transferred to a new pre-cooled 5 ml DNA low binding tube on ice and added 2 ml NIB. Nuclei were pelleted by centrifuging at 500 g× 10 min× 4 °C, resuspended in 100 µL NRB (1% BSA in PBS, 0.04 U/ µL RNase inhibitor) and filtered through a 40 µm tip strainer. 15,000 nuclei were taken out and resuspended in NRB as previous protocol described (30, 31).

10x Genomics single-nucleus RNA sequencing

We performed 10x Genomics single-nucleus RNA sequencing at USC Norris Molecular Genomics Core immediately after nuclei isolation. Single cell RNA sequencing was prepared using 10x Genomics 3' v3.1 (Cat number: 1000092) following manufacturer's protocol. Samples were parsed into single nuclei using 10x Genomics Chromium Controller and libraries were simultaneously prepared. Prepared single cell RNA sequencing libraries were sequenced on the Illumina Novaseq6000 platform at a read length of 28x 90 and read depth of 50,000 reads/ cell for 5000-7000 cells.

Single-nucleus RNA-sequencing analysis

Sequencing data was package and analyzed using 10x Genomics Cell Ranger analysis tool. The R package Seurat (V4) was used to cluster the cells in the merged matrix (32). Subclusters were annotated using the marker genes according to previous publications and online databases including “PanglaoDB” and “CellMarker” (30, 31, 33-36). GO annotation of “Biological Process” was performed using Enrichr (37).

Supplemental Figures

Supplemental Figure 1

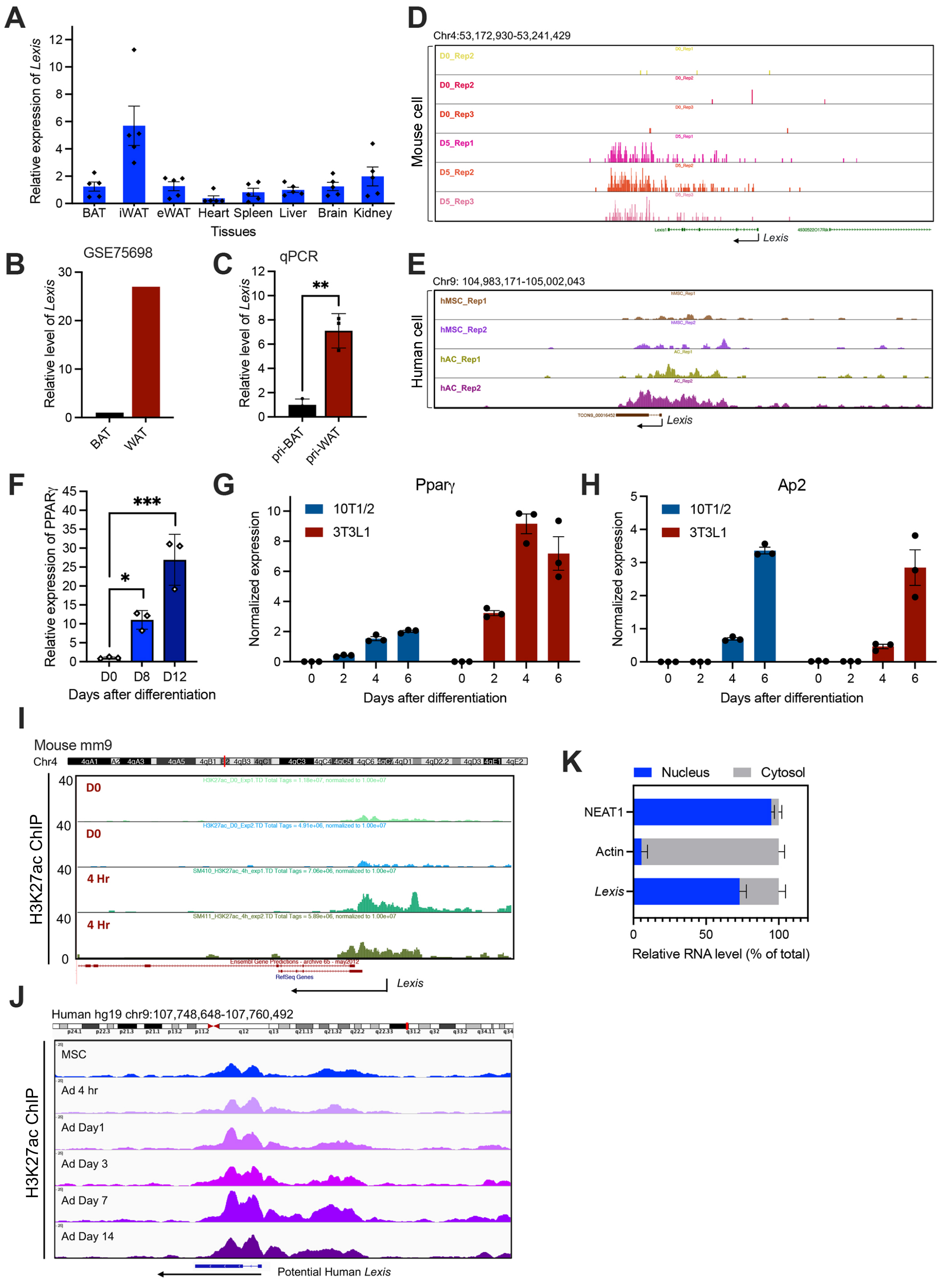

Supplemental Figure 1. *Lexis* expression in adipose tissues. (A) qPCR analysis harvested from 10 weeks old male mice on chow diet (n= 5). Data shown as mean ± SEM. **(B)** Relative levels of *Lexis* in from subcutaneous WAT (iWAT) and interscapular BAT (iBAT) published under dataset under GEO number: GSE75698. **(C)** qPCR from SVFs isolated from iWAT or iBAT (n= 3). Data shown as mean ± SD. **(D and E)** RNA-seq browser tracks near the *Lexis* region in mouse and human. **(F)** qPCR analysis in human ADMSC or differentiated adipocytes induced at Day 8 or Day 12 (n= 3). Data was shown as mean ± SD. **(G and H)** qPCR analysis in C3H10T1/2 and 3T3L1 preadipocyte cells treated with differentiation cocktail (mean ± SEM, n= 3). **(I and J)** Representative peaks near *Lexis* promoter region from ChIP-seq of H3K27ac performed in murine (I) or human cells (J). Data is from published dataset under GEO: GSE56872 and GSE95533 (mouse), GSE113253 (human). **(K)** qRT-PCR analysis of RNAs purified from fractionated compartments in C3H10T1/2 (n= 3). The nuclear paraspeckle assembly transcript 1 (NEAT1) and Actin were used as controls for the nuclear and cytoplasmic fractions, respectively. Data shown as mean ± SD. P-value calculated by unpaired t-test for supplemental Figure 1(C) or 1-way ANOVA for supplemental Figure 1(F). *: $p < 0.05$, **: $p < 0.01$, ***: $p < 0.001$.

Supplemental Figure 2

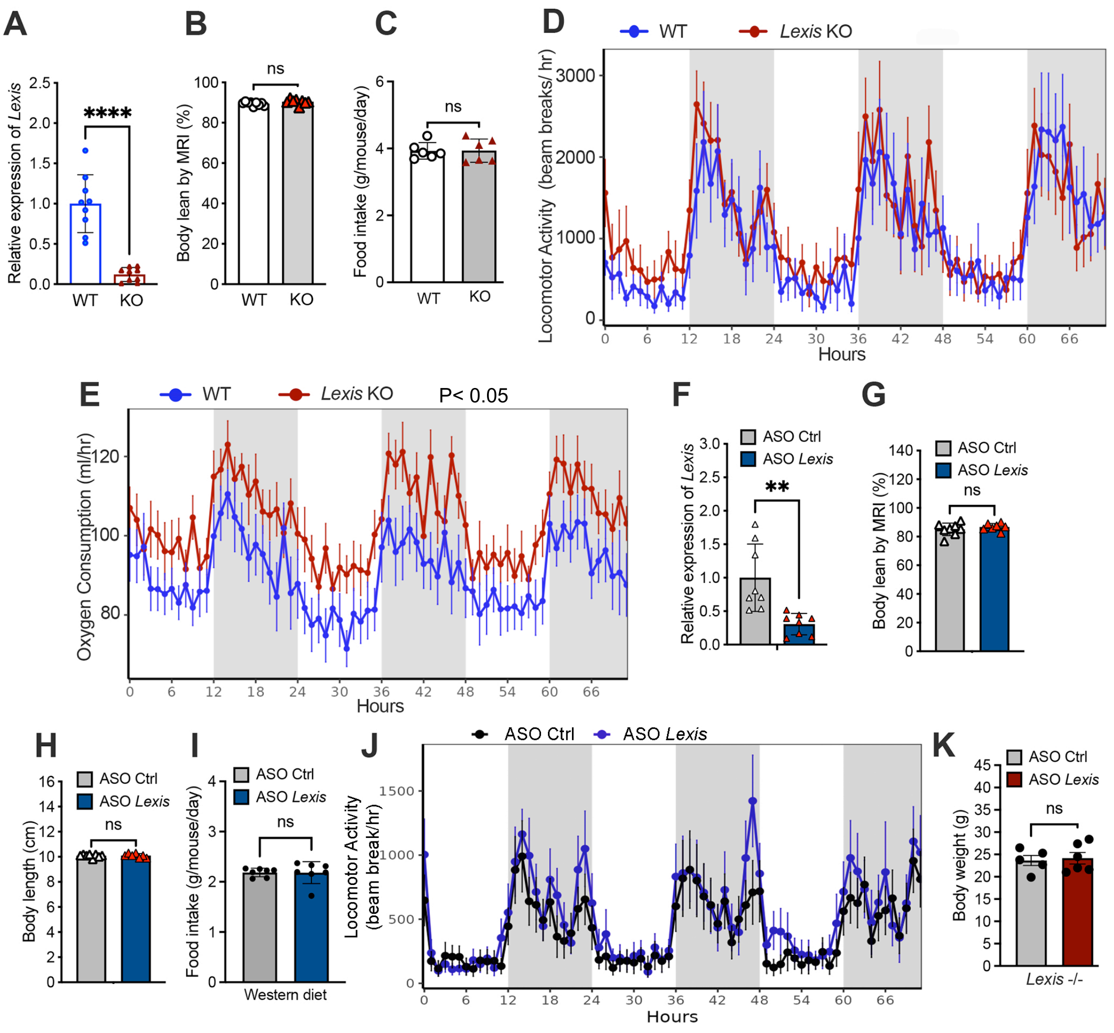

Supplemental Figure 2. Global deletion or ASO inhibition of *Lexis* lowers body weight in mice. (A) Expression of *Lexis* was detected by qRT-PCR from iWAT of *Lexis* WT or KO mice (n=9 per group). **(B)** Body lean composition of mice in Figure 2(A) was determined by EchoMRI (WT: n=9, KO: n= 8). **(C)** Food consumption from WT or *Lexis* KO mice (n=6 per group). **(D)** Local activity was detect using *Lexis* KO mice or WT mice (n= 6 per group, P= 0.4443 by ANCOVA). **(E)** VO2 was detect using *Lexis* KO mice or WT mice (n= 6 per group, P= 0.0128). **(F)** ASO knockdown efficiency in iWAT detected by qRT-PCR (n= 8 per group). **(G)** Lean composition of ASO Control or ASO *Lexis* mice from Figure 2(G) were determined by EchoMRI (Ctrl: n= 8, ASO *Lexis*: n= 7). **(H)** Body length of western diet fed ASO Control or ASO *Lexis* mice from Figure 2(G) (Ctrl: n= 8, ASO *Lexis*: n= 7). **(I)** Food consumption from ASO Control or ASO *Lexis* mice (n=7 per group). (J) Local activity was detect using *Lexis* KO mice or WT mice (n= 9 per group, P> 0.05). **(K)** Body weight in *Lexis* KO mice treated with ASO-Ctrl (n= 5) or ASO-*Lexis* (n= 6) and fed with western diet. Data was shown as mean values ± SD in Supplemental Figure 2 (A), (B), (C), (F), (G), (H) and (I), as mean values ± SEM in Supplemental Figure 2 (D), (E), (J) and (K). P-value was calculated either by unpaired t-test: supplemental Figure 2 (A), (B), (C), (F), (G), (H), (I) and (K), or by ANCOVA: supplemental Figure 2 (D), (E) and (J). **: p < 0.01, ****: p < 0.0001.

Supplemental Figure 3

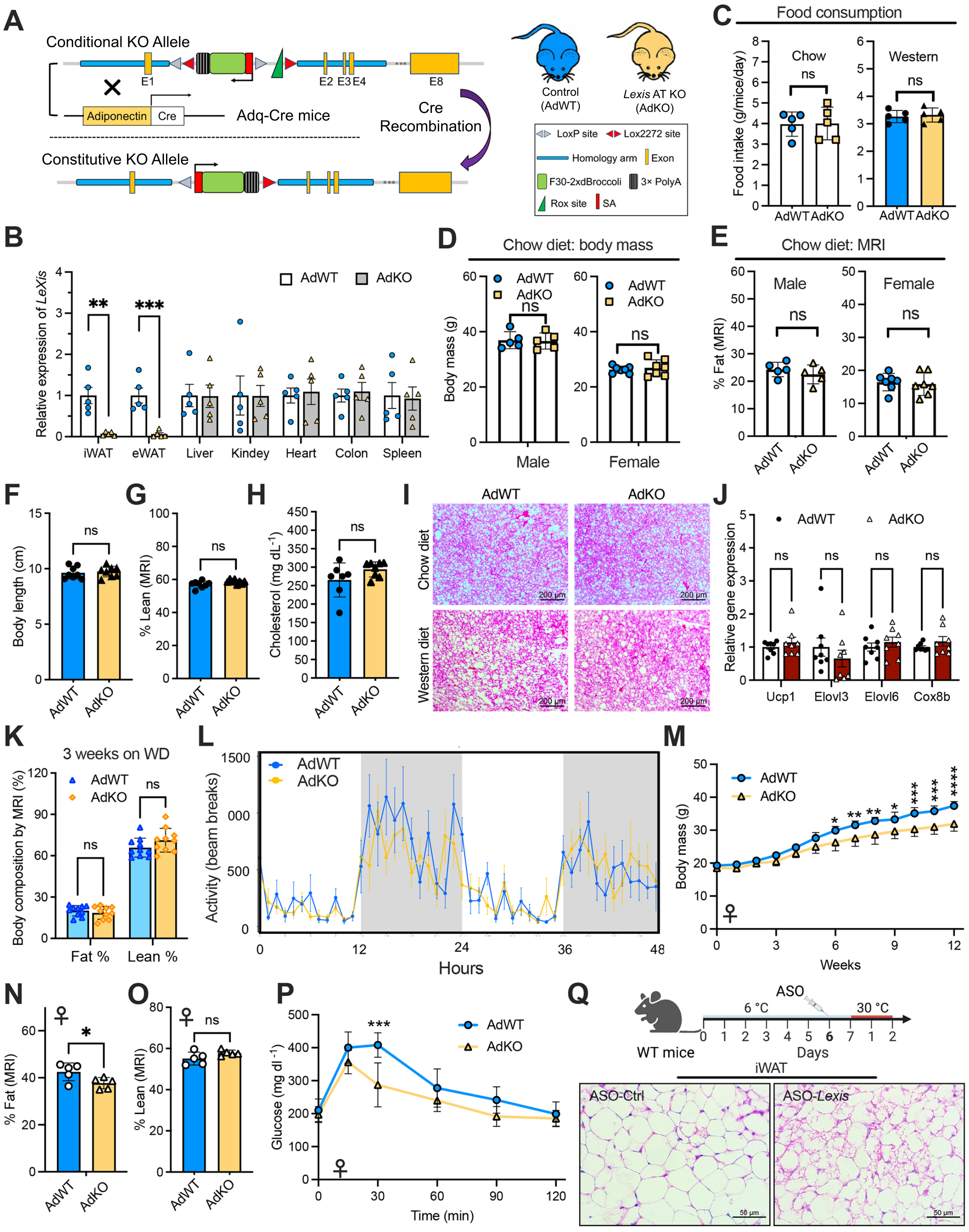

Supplemental Figure 3. Adipose-specific deletion of *Lexis* protects mice from DIO. **(A)** Schematic of tissue-specific knockout strategy **(B)** qRT-PCR from tissues (n= 5 per group) **(C)** Food consumption in male mice (n= 5 per group) **(D)** Body mass of 20-week-old male and female mice on chow diet (n= 5 male, n= 7 female) **(E)** Body fat composition of chow-fed mice in supplemental Figure 3 (D) **(F)** Body length of WD-fed male mice in Figure 3(B). **(G)** Body lean composition of WD-fed male mice in Figure 3 (B) **(H)** Serum total cholesterol from WD-fed male mice in Figure 3(B). **(I)** H&E staining of BAT from mice in with chow diet feeding or 12 weeks WD feeding. Scale bar= 200 µm. **(J)** qPCR from BAT of WD-fed mice (Male, n= 8 per group) **(K)** Body composition of WD-fed male mice before indirect calorimetry in 3H (n= 10 per group) **(L)** Locomotor activity was measured using the mice in Figure 3(H). **(M)** Body weight from female *Lexis*-AdKO and *Lexis*-AdWT mice on WD (n= 5 per group). **(N)** Fat composition from mice in supplemental Figure 3(M). **(O)** Lean composition from supplemental Figure 3 (M) **(P)** Intraperitoneal glucose tolerance test (Female, n= 5 per group) **(Q)** Temperature challenge experiment of ASO control or ASO Lexis and H&E staining of iWAT. Scale bar = 50 µm. Values are shown mean ± SD for supplemental Figure 3 (C-H), (K), (M), (N), (O) and (P). Values are shown as mean ± SEM for supplemental Figure 3 (B), (J) and (L). P-value was calculated either by unpaired t-test: supplemental Figure 3 (B-H), (J), (K), (N) and (O), or by 2-way ANOVA: supplemental Figure 3 (M) and (P). *: $p < 0.05$; **: $p < 0.01$; ***: $p < 0.001$; ****: $p < 0.0001$; ns: not significant.

Supplemental Figure 4

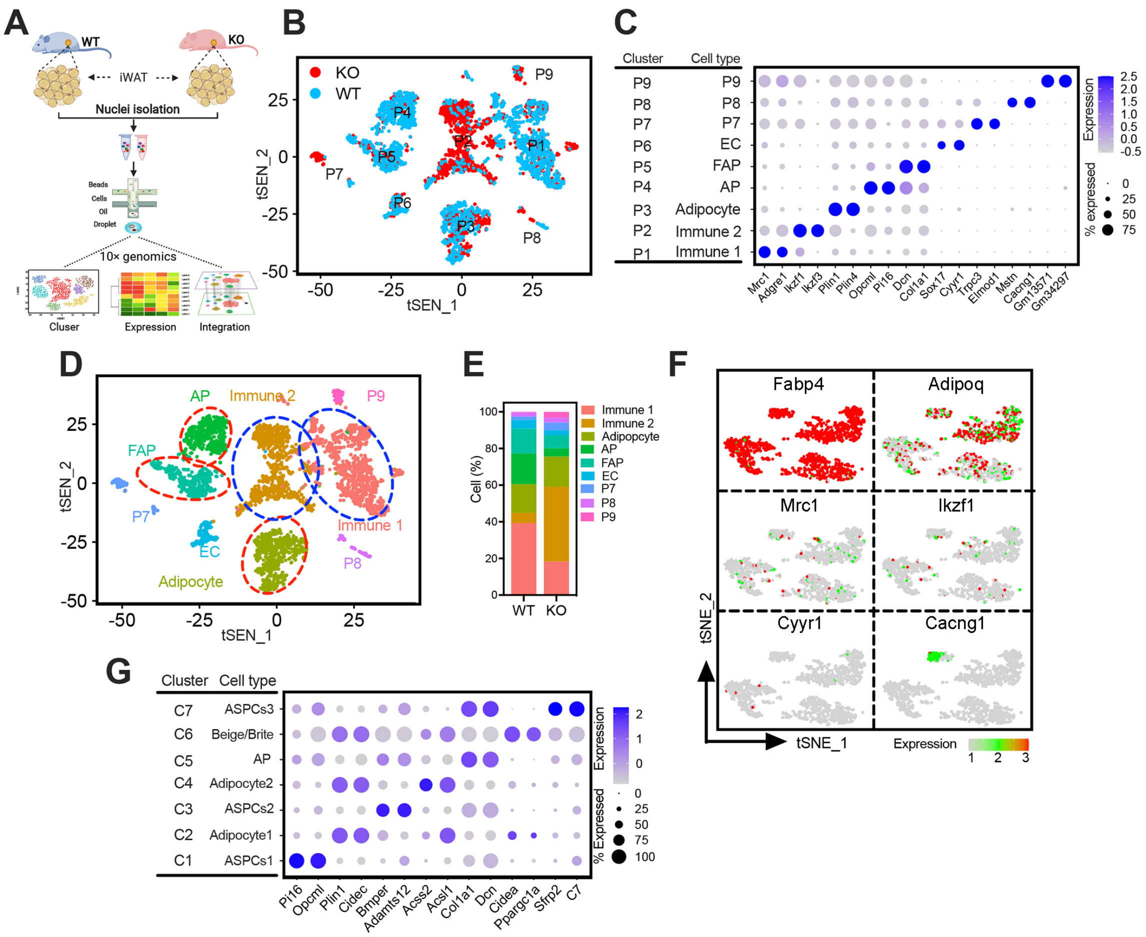

Supplemental Figure 4. snRNA-seq reveals differences in adipose cellular heterogeneity between control and *Lexis*-AdKO mice. (A) Schematic of the single nuclei RNA-seq in iWAT. For each group, nuclei were isolated and pooled from the iWAT of 3 *Lexis*-AdWT mice (WT) or *Lexis*-AdKO mice (KO) with 4 weeks WD feeding and analyzed using 10x Genomics Chromium droplet scRNA-seq. **(B)** tSNE plot of all cell types of adipose nuclei showing the difference in distribution of WT and KO cells across these clusters. **(C)** Dot plot showed average expression and percentage expression of cell marker genes in each subpopulation from all subclusters identified in iWAT. **(D)** Annotation of all subcluster identified in iWAT of *Lexis*-AdWT mice (WT) and *Lexis*-AdKO mice (KO) based on cell marker genes (see supplemental Table 1 for marker details). **(E)** The fraction of each cell type relative to the total number of nuclei in iWAT of *Lexis*-AdWT mice (WT) and *Lexis*-AdKO mice (KO). **(F)** tSNE plots highlighting the expression of Fabp4, Adipoq, Mrc1, Ikzf1, Cyyr1 and Cacng1 in adipocyte subclusters. **(G)** Dot plot showing average expression and percentage expression of cell marker genes in each subpopulation from all adipocyte subclusters.

Supplemental Figure 5

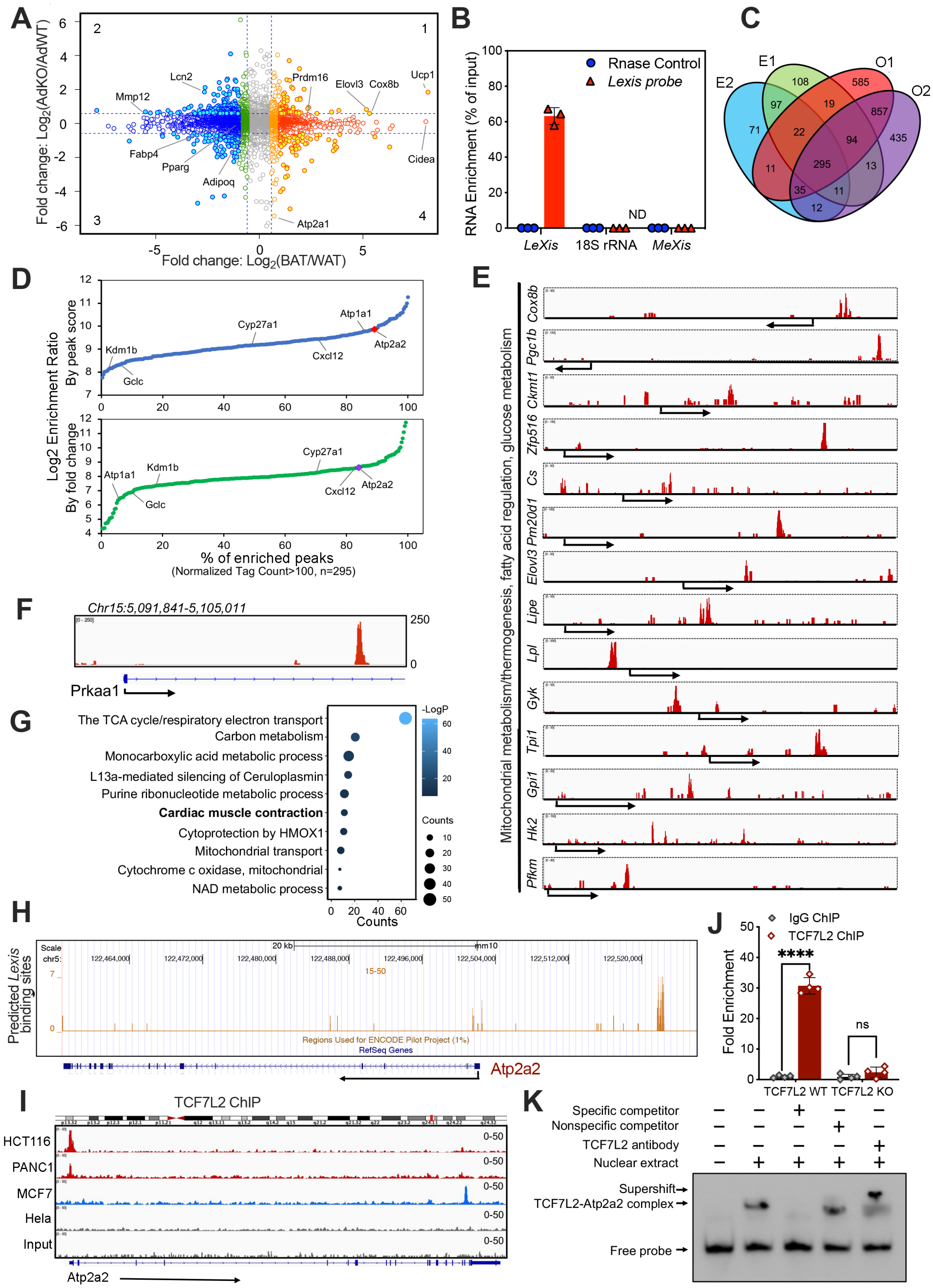

Supplemental Figure 5. Systemic discovery of *Lexis* interactome. (A) Gene expression signatures of AdWT and *Lexis* AdKO iWAT and reference BAT and WAT from public datasets GSE8044 (Seale et al., 2007). **(B)** RNA enrichment with biotin labeled probes retrieving *Lexis* with or without RNase treatment, determined by qRT-qPCR and calculated as percentage of Input (n= 3 per group). 18s rRNA and *Mexis* are used as negative control. Data was shown as mean ± SD. **(C)** Venn diagram analysis of significantly enriched genes among 4 probe sets (Cutoff: P-value<0.01, Peaks Score [FPS]>100). **(D)** An average enrichment profile is shown for common genes in Supplemental Figure 5 (C) calculated by either "peak score" (upper panel) or by "fold change" (lower panel). **(E)** Peaks of *Lexis* are shown for genes related to mitochondrial metabolism, thermogenesis, fatty acid regulation and glucose metabolism. **(F)** *Lexis*-dependent contact on Ca^{2+} related gene *Prkaa1*. **(G)** Top 10 enriched pathways analyzed by Metascape using the data from published dataset ArrayExpress: E-MTAB-4085. Differentially expressed genes were obtained by comparing the group of "Prdm16 Tg × $Ucp1^{-/-}$" to "$Ucp1^{-/-}$". **(H)** Prediction of binding sites of *Lexis* on Atp2a2 genomic loci by software "LongTarget". **(I)** TCF7L2 ChIP-seq peaks on Atp2a2 genomic loci from different human cell lines. Peaks were visualized using datasets under GEO numbers: GSM782123, GSM816437, GSM816438, GSM816436, GSM817344. **(J)** TCF7L2 ChIP at *Axin2* in TCF7L2 WT or TCF7L2 KO preadipocyte (n= 4 per group). Data shown as mean ± SD. P-value was calculated by unpaired t-test. ****: $p < 0.0001$; ns: not significant. **(K)** Biotin-labelled EMSA performed using preadipocyte nuclear extracts. Excess unlabeled probes used as specific competitor.

Supplemental Figure 6

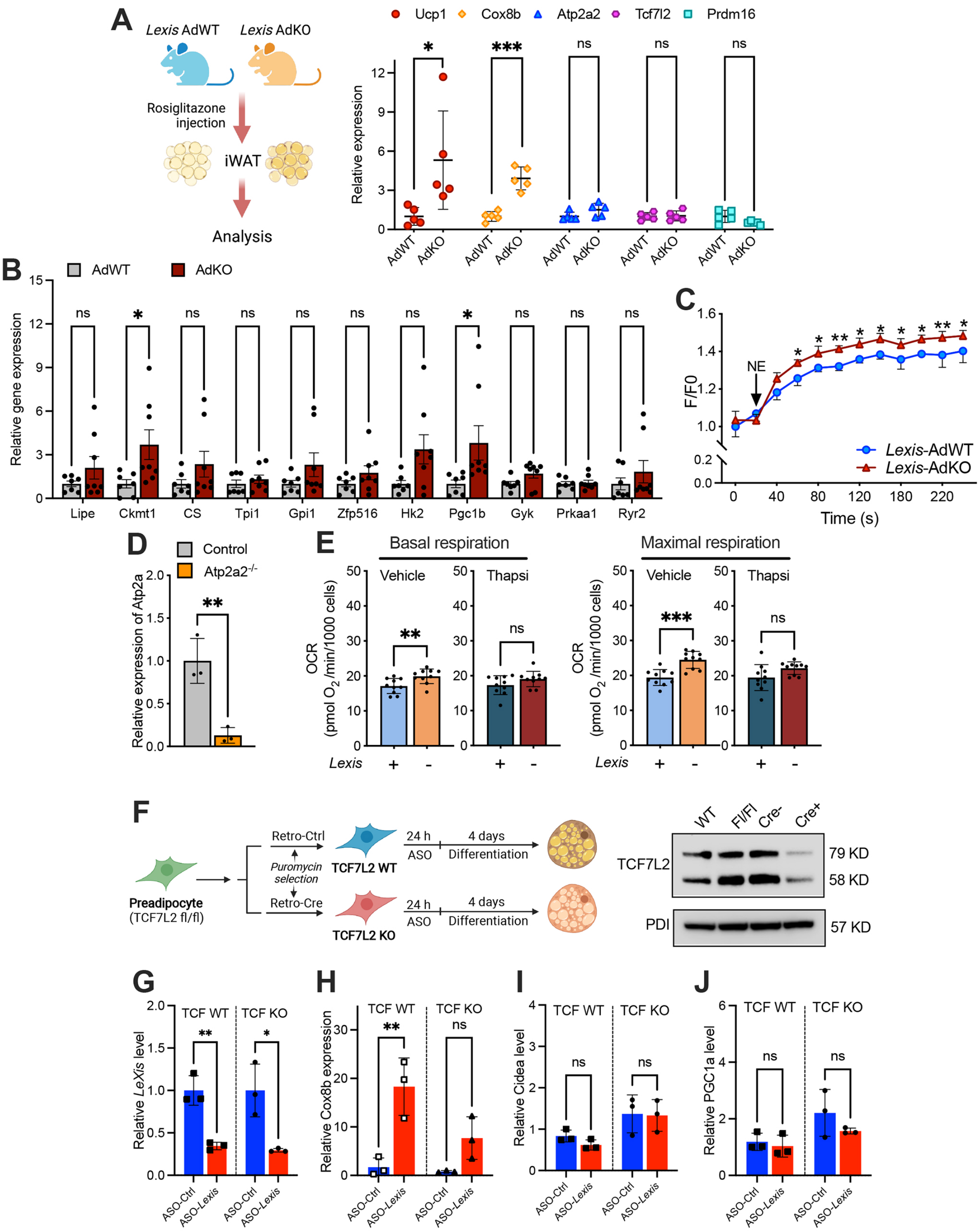

Supplemental Figure 6. *Lexis* integrates UCP-dependent and independent thermogenic pathways. (A) Gene expression by qRT-PCR in iWAT of the mice with rosiglitazone treatment (n= 5 per group). Rosiglitazone IP injected at the dose of 5 mg/kg for 7 days. **(B)** qPCR in iWAT of *Lexis* WT or KO mice from Figure 3 (K) (n= 7 for AdWT, n= 8 for AdKO). Data was shown as mean ± SEM. **(C)** Ca^{2+} level induced by NE was detected in SVF isolated from iWAT of *Lexis*-AdWT or *Lexis*-AdKO mice (n= 4 per group). **(D)** qPCR detection of Atp2a2 from Atp2a2 WT or Atp2a2 CRISPR KO preadipocyte (n= 3 per group). Data was shown as mean ± SD. **(E)** Immortalized *Lexis* WT or KO preadipocytes induced by browning cocktail for 5 days. Adipocytes were treated with 2 μM Thapsigargin for 1 hour. Basal and maximal respiration was shown (n=10 per group). **(F)** Schematic for generation of TCF7L2 KO preadipocyte and treatment related to Figure 6 (H) and (I), supplemental Figure 6 (G-J). **(G)** *Lexis* levels determined by qRT-PCR in TCF7L2 WT or TCF7L2 KO preadipocyte (n= 3 per group). **(H-J)** Gene expression of Cox8b, Cidea and PGC1a by qRT-PCR form TCF7L2 WT or KO adipocyte with ASO-Ctrl or ASO-*Lexis* transfection (n= 3 per group). All error bars indicate SD other than supplemental Figure 6 (B) in which data was shown as mean ± SEM. P-value was calculated by unpaired t-test: supplemental Figure 6 (A), (B), (D), (E), (G), (H), (I) and (J). 2-way ANOVA supplemental Figure 6 (C). *: $p < 0.05$, **: $p < 0.01$, ***: $p < 0.001$.

Supplemental Tables

Supplemental Table 1. Gene markers for each single-nucleus RNA-seq cluster

Cluster	Sequence	Reference
**All clusters:**		
P1: Immune_1	Mrc1, Adgre1	Adgre1: (31); Mrc1: Panglao database; CellMarker.
P2: Immune_2	Ikzf1, Ikzf3	Panglao database; CellMarker.
P3: Adipocyte	Plin1, Plin4	(31, 38); Panglao database; CellMarker.
P4: AP	Opcml, Pi16, Dpp4	(38).
P5: FAP	Dcn, Pdgfa, Col1a1	(31).
P6: EC	Sox17, Cyyr1	(39, 40); CellMarker.
P7	Trpc3, Elmod1	NA
P8	Mstn, Cacng1	NA
P9	Gm13571, Gm34297	NA
**Adipocyte clusters**		
C1: ASPCs 1	Pi16, Opcml	(38).
C2: Adipocyte 1	Plin1, Cidec	(38, 41); Panglao database; CellMarker.
C3: ASPCs 2	Bmper, Adamts12, Gpm6b, Gria3	(41); CellMarker.
C4: Adipocyte 2	Acss2, Acsl1, Cidec	(42).
C5: AP2	Col1a1, Dcn, Pdgfra	(31).
C6: Beige/Brite	Cidea, Ppargc1a	(42); (43).
C7: ASPCs 3	Frmpd4, Fmo6	(41).

Supplemental Table 2. Key resources table

REAGENT or RESOURCE	SOURCE	Cat#
**Antibodies**		
PDI (C81H6)	Cell signaling	3501S
TCF4/TCF7L2 (C48H11)	Cell signaling	2569S
**Chemicals, Peptides, and Recombinant Proteins**		
3-isobutyl-1-methylxanthine	Sigma	I-7018
Dexamethasone	Sigma	D-2915
Rosiglitazone	Sigma	R-2408
3,3′,5-Triiodo-L-thyronine (T3)	Sigma	T-2877
Indomethacin	Sigma	I-7378
Puromycin	Sigma	P9620
Collagenase D	Roche	11088882001
Collagenase B	Roche	11088831001
Dispase II	Roche	04942078001
Insulin	ThermoFisher Scientific	12585-014
CL316,243	Sigma	C5976
Stellaris® RNA FISH Hybridization Buffer	LGC Biosearch Technologies	SMF-HB1-10
Stellaris® RNA FISH Wash Buffer A	LGC Biosearch Technologies	SMF-WA1-60
Stellaris® RNA FISH Wash Buffer B	LGC Biosearch Technologies	SMF-WB1-20
Fluo-8 AM, green-fluorescent calcium binding dye	Abcam	Ab142773
ProLong™ Diamond Antifade Mountant with DAPI	ThermoFisher Scientific	P36966
DMEM	CORNING	10-013-CM
FBS	Omega Scientific	FB-11
DMEM/F12 Glutamax	ThermoFischer Scientific	10565-018
Penicillin/Streptomycin	CORNING	MT-30-002-CI
**Kits**		
10x Genomics 3' v3.1	10x Genomics	1000092
LightShift EMSA Kit	ThermoFisher Scientific	89880
LightShift™ Poly (dI-dC)	ThermoFisher Scientific	20148E
BCA protein assay kit	ThermoFisher Scientific	23225
Fluo-8 Calcium Flux Assay Kit - No Wash	Abcam	Ab112129
NE-PER™ Nuclear and Cytoplasmic Extraction Reagents	ThermoFisher Scientific	78833
**Enzymes and inhibitors**		
Protector RNase Inhibitor (For snRNA-seq)	Millipore Sigma	3335402001
Invitrogen™ RNaseOUT™ (For RT and ChIRP)	Invitrogen	10777019
Protease Inhibitor Cocktail	Millipore Sigma	11836145001
Thapsigargin	Millipore Sigma	T9033
**Mice and Cell lines**		
Lexis global KO mice	Sallam et al., 2016	N/A
Lexis^{Flox} mice	This Paper	N/A
Ucp1 KO mice	Jackson Laboratory	003124
TCF7L2 KO immortalized preadipocytes	Geoghegan et al., 2019	N/A

C3H10T1/2	ATCC	CCL-226™
3T3L1	ATCC	CL-173
Adipose-Derived Mesenchymal Stem Cells	ATCC	PCS-500-011
**Software and Algorithms**		
HOMER	Heinz et al., 2010	N/A
MACS2	Zhang et al., 2008	N/A
Pscan	Zambelliet al., 2013	N/A
Samtools	Li et al., 2009	N/A
SeqMonk	Babraham Bioinformatics	N/A
Bowtie2	Langmead and Salzberg, 2012	N/A
DESeq2	Love et al., 2014	N/A
Seurat (V4)	Hao et al., 2021	N/A
Cell Ranger	10x Genomics	N/A
LongTarget	Liu et al., 2017	N/A
motifStack	Ou et al., 2018	N/A
ImageJ	https://imagej.nih.gov/ij/	N/A
Prism 9	GraphPad	N/A
**Diets**		
High fat diet	Research Diets	D12492
Western diet	Research Diets	D12079B
**Other**		
Cell culture chamber slide	Nest Scientific	230114

Supplemental Table 3. Sequences of Primers, Probes and Oligonucleotides*

ID	Sequence	Application
**ChIRP/ChIP-qPCR:**		
Atp2a2-Peak3_Forward	GCCTGCCAACAGTTTGGTTT	*Lexis* ChIRP-qPCR
Atp2a2-Peak3_Reverse	CCGAGCTGACCCTGGTCTAT	*Lexis* ChIRP-qPCR
Atp2a2-S1_Forward	ATGCTAGGTGGCTTGGCTTT	TCF7L2 ChIP-qPCR
Atp2a2-S1_Reverse	AGGCTCCTTCTCTCGCATTG	TCF7L2 ChIP-qPCR
Atp2a2-S2_Forward	TGTCTGTGATGCTCACTGGG	TCF7L2 ChIP-qPCR
Atp2a2-S2_Reverse	AGGGAAAAACTGGGAACCCG	TCF7L2 ChIP-qPCR
NC_Forward	GTTCTTTGCCAGCTGAAGGC	TCF7L2 ChIP-qPCR
NC_Reverse	CCTCTCTCCTCCCAGTGACA	TCF7L2 ChIP-qPCR
**RNA FISH**		
Lexis_18_1	ctctagctccggagtcag	RNA FISH
Lexis_18_2	tctcgctcgaatgtgctc	RNA FISH
Lexis_18_3	ttgtttgcacccacgtag	RNA FISH
Lexis_18_4	cccgggcagaaactggag	RNA FISH
Lexis_18_5	agttgttagatccggctc	RNA FISH
Lexis_18_6	aggtaggggcttgaactc	RNA FISH
Lexis_18_7	agagaccacaggtcggtc	RNA FISH
Lexis_18_8	tggggagcattgtccaac	RNA FISH
Lexis_18_9	ggtattctgtttgcacct	RNA FISH
Lexis_18_10	agagacaaagcccggtca	RNA FISH
Lexis_18_11	cctccatactgctgcaaa	RNA FISH
Lexis_18_12	ccaaagccttgctccaac	RNA FISH
Lexis_18_13	taagggctgttttgtgcc	RNA FISH
Lexis_18_14	ccaagtgctcaagtgtcc	RNA FISH
Lexis_18_15	agagaggtgccatgaagc	RNA FISH
Lexis_18_16	aggtggcactgagagtgt	RNA FISH
**EMSA**		
ATP2a2_EMSA_1F	tcttccaaagtgtagacattaaaaccctttaatccattgcactagattg	EMSA
ATP2a2_EMSA_1R	caatctagtgcaaatggattaaagggttttaatgtctacactttggaaga	EMSA

*Most of the qRT-PCR primers used in this study are from our previous publications (1, 2, 4, 27, 28, 44, 45). The primers in table are for ChIP-qPCR and ChIRP-qPCR are generated in this study.

References

1. Geoghegan G, Simcox J, Seldin MM, Parnell TJ, Stubben C, Just S, et al. Targeted deletion of Tcf7l2 in adipocytes promotes adipocyte hypertrophy and impaired glucose metabolism. *Mol Metab.* 2019;24:44-63.
2. Rajbhandari P, Thomas BJ, Feng AC, Hong C, Wang J, Vergnes L, et al. IL-10 Signaling Remodels Adipose Chromatin Architecture to Limit Thermogenesis and Energy Expenditure. *Cell.* 2018;172(1-2):218-33 e17.
3. Wang J, Rajbhandari P, Damianov A, Han A, Sallam T, Waki H, et al. RNA-binding protein PSPC1 promotes the differentiation-dependent nuclear export of adipocyte RNAs. *J Clin Invest.* 2017;127(3):987-1004.
4. Sallam T, Jones MC, Gilliland T, Zhang L, Wu X, Eskin A, et al. Feedback modulation of cholesterol metabolism by the lipid-responsive non-coding RNA LeXis. *Nature.* 2016;534(7605):124-8.
5. Mina AI, LeClair RA, LeClair KB, Cohen DE, Lantier L, and Banks AS. CalR: A Web-Based Analysis Tool for Indirect Calorimetry Experiments. *Cell Metab.* 2018;28(4):656-66 e1.
6. Acin-Perez R, Benador IY, Petcherski A, Veliova M, Benavides GA, Lagarrigue S, et al. A novel approach to measure mitochondrial respiration in frozen biological samples. *EMBO J.* 2020;39(13):e104073.
7. Osto C, Benador IY, Ngo J, Liesa M, Stiles L, Acin-Perez R, et al. Measuring Mitochondrial Respiration in Previously Frozen Biological Samples. *Curr Protoc Cell Biol.* 2020;89(1):e116.
8. Pandya-Jones A, and Black DL. Co-transcriptional splicing of constitutive and alternative exons. *RNA.* 2009;15(10):1896-908.
9. Church CD, Berry R, and Rodeheffer MS. Isolation and study of adipocyte precursors. *Methods Enzymol.* 2014;537:31-46.
10. Pearson S, Loft A, Rajbhandari P, Simcox J, Lee S, Tontonoz P, et al. Loss of TLE3 promotes the mitochondrial program in beige adipocytes and improves glucose metabolism. *Genes Dev.* 2019;33(13-14):747-62.
11. Ikeda K, Kang Q, Yoneshiro T, Camporez JP, Maki H, Homma M, et al. UCP1-independent signaling involving SERCA2b-mediated calcium cycling regulates beige fat thermogenesis and systemic glucose homeostasis. *Nat Med.* 2017;23(12):1454-65.
12. Pertea M, Kim D, Pertea GM, Leek JT, and Salzberg SL. Transcript-level expression analysis of RNA-seq experiments with HISAT, StringTie and Ballgown. *Nat Protoc.* 2016;11(9):1650-67.
13. Love MI, Huber W, and Anders S. Moderated estimation of fold change and dispersion for RNA-seq data with DESeq2. *Genome Biol.* 2014;15(12):550.
14. Zhou Y, Zhou B, Pache L, Chang M, Khodabakhshi AH, Tanaseichuk O, et al. Metascape provides a biologist-oriented resource for the analysis of systems-level datasets. *Nat Commun.* 2019;10(1):1523.
15. Hao RH, Guo Y, Wang C, Chen F, Di CX, Dong SS, et al. Lineage-specific rearrangement of chromatin loops and epigenomic features during adipocytes and osteoblasts commitment. *Cell Death Differ.* 2022;29(12):2503-18.

16. Liao Y, Smyth GK, and Shi W. featureCounts: an efficient general purpose program for assigning sequence reads to genomic features. *Bioinformatics.* 2014;30(7):923-30.
17. Frankish A, Diekhans M, Jungreis I, Lagarde J, Loveland JE, Mudge JM, et al. Gencode 2021. *Nucleic Acids Res.* 2021;49(D1):D916-D23.
18. Chu C, Quinn J, and Chang HY. Chromatin isolation by RNA purification (ChIRP). *J Vis Exp.* 2012(61).
19. Mumbach MR, Granja JM, Flynn RA, Roake CM, Satpathy AT, Rubin AJ, et al. HiChIRP reveals RNA-associated chromosome conformation. *Nat Methods.* 2019;16(6):489-92.
20. Langmead B, and Salzberg SL. Fast gapped-read alignment with Bowtie 2. *Nat Methods.* 2012;9(4):357-9.
21. Liu T. Use model-based Analysis of ChIP-Seq (MACS) to analyze short reads generated by sequencing protein-DNA interactions in embryonic stem cells. *Methods Mol Biol.* 2014;1150:81-95.
22. Zhang Y, Liu T, Meyer CA, Eeckhoute J, Johnson DS, Bernstein BE, et al. Model-based analysis of ChIP-Seq (MACS). *Genome Biol.* 2008;9(9):R137.
23. Ou J, Wolfe SA, Brodsky MH, and Zhu LJ. motifStack for the analysis of transcription factor binding site evolution. *Nat Methods.* 2018;15(1):8-9.
24. Zambelli F, Pesole G, and Pavesi G. PscanChIP: Finding over-represented transcription factor-binding site motifs and their correlations in sequences from ChIP-Seq experiments. *Nucleic Acids Res.* 2013;41(Web Server issue):W535-43.
25. He S, Zhang H, Liu H, and Zhu H. LongTarget: a tool to predict lncRNA DNA-binding motifs and binding sites via Hoogsteen base-pairing analysis. *Bioinformatics.* 2015;31(2):178-86.
26. Liu H, Shang X, and Zhu H. LncRNA/DNA binding analysis reveals losses and gains and lineage specificity of genomic imprinting in mammals. *Bioinformatics.* 2017;33(10):1431-6.
27. Villanueva CJ, Waki H, Godio C, Nielsen R, Chou WL, Vargas L, et al. TLE3 is a dual-function transcriptional coregulator of adipogenesis. *Cell Metab.* 2011;13(4):413-27.
28. Villanueva CJ, Vergnes L, Wang J, Drew BG, Hong C, Tu Y, et al. Adipose subtype-selective recruitment of TLE3 or Prdm16 by PPARgamma specifies lipid storage versus thermogenic gene programs. *Cell Metab.* 2013;17(3):423-35.
29. Iyer LM, Nagarajan S, Woelfer M, Schoger E, Khadjeh S, Zafiriou MP, et al. A context-specific cardiac beta-catenin and GATA4 interaction influences TCF7L2 occupancy and remodels chromatin driving disease progression in the adult heart. *Nucleic Acids Res.* 2018;46(6):2850-67.
30. Van Hauwaert EL, Gammelmark E, Sarvari AK, Larsen L, Nielsen R, Madsen JGS, et al. Isolation of nuclei from mouse white adipose tissues for single-nucleus genomics. *STAR Protoc.* 2021;2(3):100612.
31. Sarvari AK, Van Hauwaert EL, Markussen LK, Gammelmark E, Marcher AB, Ebbesen MF, et al. Plasticity of Epididymal Adipose Tissue in Response to Diet-Induced Obesity at Single-Nucleus Resolution. *Cell Metab.* 2021;33(2):437-53 e5.
32. Hao Y, Hao S, Andersen-Nissen E, Mauck WM, 3rd, Zheng S, Butler A, et al. Integrated analysis of multimodal single-cell data. *Cell.* 2021;184(13):3573-87 e29.

33. Franzen O, Gan LM, and Bjorkegren JLM. PanglaoDB: a web server for exploration of mouse and human single-cell RNA sequencing data. *Database (Oxford).* 2019;2019.
34. Zhang X, Lan Y, Xu J, Quan F, Zhao E, Deng C, et al. CellMarker: a manually curated resource of cell markers in human and mouse. *Nucleic Acids Res.* 2019;47(D1):D721-D8.
35. Schwalie PC, Dong H, Zachara M, Russeil J, Alpern D, Akchiche N, et al. A stromal cell population that inhibits adipogenesis in mammalian fat depots. *Nature.* 2018;559(7712):103-+.
36. Sun W, Dong H, Balaz M, Slyper M, Drokhlyansky E, Colleluori G, et al. snRNA-seq reveals a subpopulation of adipocytes that regulates thermogenesis. *Nature.* 2020;587(7832):98-102.
37. Kuleshov MV, Jones MR, Rouillard AD, Fernandez NF, Duan Q, Wang Z, et al. Enrichr: a comprehensive gene set enrichment analysis web server 2016 update. *Nucleic Acids Res.* 2016;44(W1):W90-7.
38. Merrick D, Sakers A, Irgebay Z, Okada C, Calvert C, Morley MP, et al. Identification of a mesenchymal progenitor cell hierarchy in adipose tissue. *Science.* 2019;364(6438).
39. Xie T, Wang Y, Deng N, Huang G, Taghavifar F, Geng Y, et al. Single-Cell Deconvolution of Fibroblast Heterogeneity in Mouse Pulmonary Fibrosis. *Cell Rep.* 2018;22(13):3625-40.
40. Zhang L, Jambusaria A, Hong Z, Marsboom G, Toth PT, Herbert BS, et al. SOX17 Regulates Conversion of Human Fibroblasts Into Endothelial Cells and Erythroblasts by Dedifferentiation Into CD34(+) Progenitor Cells. *Circulation.* 2017;135(25):2505-23.
41. Schwalie PC, Dong H, Zachara M, Russeil J, Alpern D, Akchiche N, et al. A stromal cell population that inhibits adipogenesis in mammalian fat depots. *Nature.* 2018;559(7712):103-8.
42. Rajbhandari P, Arneson D, Hart SK, Ahn IS, Diamante G, Santos LC, et al. Single cell analysis reveals immune cell-adipocyte crosstalk regulating the transcription of thermogenic adipocytes. *Elife.* 2019;8.
43. Song A, Dai W, Jang MJ, Medrano L, Li Z, Zhao H, et al. Low- and high-thermogenic brown adipocyte subpopulations coexist in murine adipose tissue. *J Clin Invest.* 2020;130(1):247-57.
44. Sallam T, Jones M, Thomas BJ, Wu X, Gilliland T, Qian K, et al. Transcriptional regulation of macrophage cholesterol efflux and atherogenesis by a long noncoding RNA. *Nat Med.* 2018;24(3):304-12.
45. Moore TM, Cheng L, Wolf DM, Ngo J, Segawa M, Zhu X, et al. Parkin regulates adiposity by coordinating mitophagy with mitochondrial biogenesis in white adipocytes. *Nat Commun.* 2022;13(1):6661.

Full unedited gel for Supplemental Figure 5K

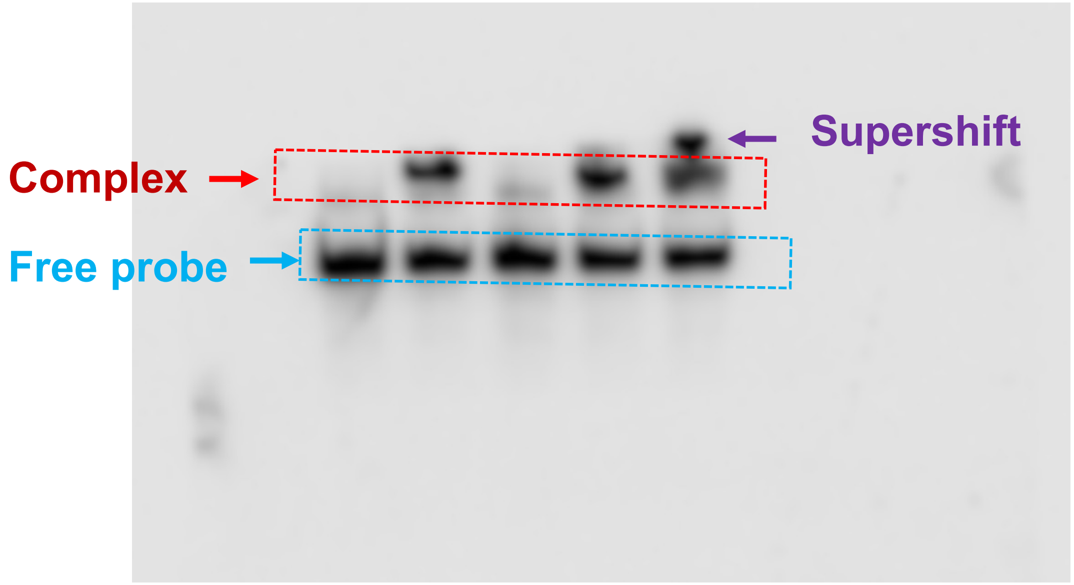

Full unedited gene for Supplementary Figure 6F

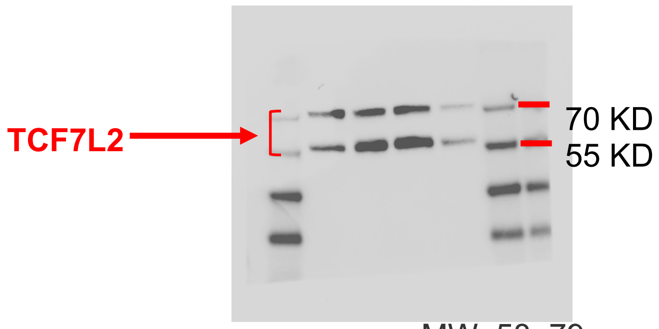

MW: 58, 79

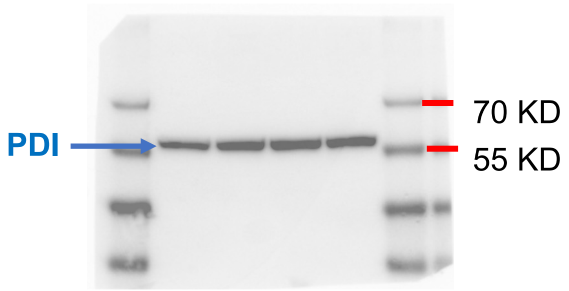

MW: 57

Protein ladder: ThermoFisher, CAT#: 26619
